# Coupled fibromodulin and SOX2 signaling as a critical regulator of metastatic outgrowth in melanoma

**DOI:** 10.1007/s00018-022-04364-5

**Published:** 2022-06-23

**Authors:** Victor O. Oria, Hongyi Zhang, Christopher R. Zito, Chetan K. Rane, Xian-Yong Ma, Olivia K. Provance, Thuy T. Tran, Adebowale Adeniran, Yuval Kluger, Mario Sznol, Marcus W. Bosenberg, Harriet M. Kluger, Lucia B. Jilaveanu

**Affiliations:** 1 Section of Medical Oncology, Department of Medicine, Yale University School of Medicine, 333 Cedar Street, SHM234E, New Haven, CT 06520 USA; 2 Biotech Research and Innovation Centre (BRIC), Faculty of Health and Medical Sciences, University of Copenhagen, 2200 Copenhagen, Denmark; 3 Department of Microbiology and Immunology, School of Medicine, Jinan University, Guangzhou, Guangdong China; 4 Department of Biology, School of Arts, Sciences, Business, and Education, University of Saint Joseph, West Hartford, CT USA; 5 Department of Pathology, Yale University School of Medicine, New Haven, CT USA; 6 Department of Dermatology, Yale University School of Medicine, New Haven, CT USA

**Keywords:** FMOD, Extracellular matrix, Brain metastasis, Vasculogenic mimicry, Hippo pathway

## Abstract

We aimed to study mechanisms controlling metastatic outgrowth of melanoma into clinically relevant lesions, a critical process responsible for the majority of melanoma deaths. To this end, we developed novel in vivo models and identified molecular events that can be ascribed to their distinct phenotypes, indolent or highly metastatic. Induction of a proliferative state at distant sites was associated with high levels of the stem-like/progenitor marker, SOX2, and required the upregulation of FMOD, an extracellular matrix component, which modulates tumor–stroma interactions. Functional studies revealed a possible link between FMOD and SOX2; dual FMOD and SOX2 silencing nearly abolished brain metastasis and had a similar effect on distant metastasis to other sites. Our in vitro data suggests that FMOD and SOX2 cooperation plays an important role in tumor vasculogenic mimicry. Furthermore, we found that FMOD and SOX2 functional roles might converge at the activation of transcriptional co-factors YAP and TAZ, possibly via crosstalk with the tumor suppressor Hippo pathway. Finally, high expression of both genes in patient specimens predicted early development of brain metastasis. Thus, our study identifies FMOD and SOX2 cooperation as a novel regulatory mechanism that might be linked functionally to melanoma metastatic competence.

**Supplementary Information:**

The online version contains supplementary material available at 10.1007/s00018-022-04364-5.

## Introduction

The main cause of death from melanoma is the uncontrolled growth of micrometastases into clinically apparent and difficult to eradicate macrometastases. Our understanding of the mechanisms that trigger or sustain metastatic initiation and outgrowth in melanoma is limited primarily due to the paucity of adequate preclinical models for functional studies and limited access to relevant clinical specimens. A critical step for metastatic initiation and progression is the establishment of supportive microenvironments, which depends in part on specific interactions between metastasizing cancer cells and host tissue stroma at various anatomical locations [1, 2]. The tumor microenvironment (TME) consists of an insoluble extracellular matrix (ECM), a stroma composed of fibroblasts, adipocytes, endothelial, immune, and inflammatory cells, and functions as an essential source for growth, survival, motility, and angiogenic factors. The ECM consists of a dynamic network of macromolecules including fibrillar proteins, glycoproteins, and proteoglycans that are synthesized and released by surrounding cells [3]. Notably, ECM composition is distinct in each tissue type and is differentially altered with metastasis [4]. In addition to general functions such as structure and stability, ECM proteins can activate autocrine and paracrine cell signaling pathways regulating migration, survival, and the growth of tumor or resident cells. Altered expression of small leucine-rich proteoglycans (SLRPs) such as decorin, biglycan, lumican, versican, and fibromodulin in tumors impacts growth factor activity and matrix assembly and can have either pro- or anti-tumorigenic effects [3, 5, 6]. Tumor cells can themselves secrete various ECM factors and alter the secretory activity of local stromal cells to induce a growth-permissive TME [7].

The fate of disseminated cancer cells, survival, and differentiation at secondary sites are highly dependent on the microenvironmental conditions [1, 2]. Once cancer cells extravasate and seed at a secondary site parenchyma, they can arrest or develop as perivascular lesions depending on specific signals from the metastatic niche, formed by tumor cells in cooperation with stromal cells [8]. Metastatic niches can play a key role in supporting the survival and self-renewal capacity of disseminated cancer cells [2]. The stem cell-like property of self-renewal, or stemness, seems to be induced early after infiltration of secondary tissues and is preserved throughout colonization by both cell-autonomous mechanisms and microenvironmental signals [2, 9]. Several specific ECM molecules or ECM-interacting proteins are essential components of metastatic niches during the initiation of metastatic colonization. For example, Tenascin C (TNC) and periostin (POSTN) play important roles in both the survival and maintenance of stem cell functions of disseminated breast cancer cells and are required for metastatic outgrowth [2]. Additional studies have shown that microenvironments rich in type I collagen or fibronectin promote metastatic outgrowth [1]. Similarly, tumor cell-derived collagen-crosslinking enzymes LOX and PLOD2 promote metastasis through stiffening the ECM, by mediating integrin–focal adhesion signaling [1].

Nevertheless, self-renewal capacity is important but insufficient for metastatic initiation and is usually coupled with the other mechanisms, such as niche activation and/or angiogenic switch, which remain largely understudied [2, 9]. Thrombospondin 2 (THBS2), for example, a mesenchymal state-dependent tumor secreted ECM molecule promoted stromal niche activation via integrin-mediated signaling in the early phase of colonization of metastasis-initiating breast cancer cells [9]. Stromal activation in turn induced cells to undergo reversion to a more epithelium-like phenotype, which often induces the proliferation of disseminated tumor cells into overt metastases [2, 9]. Similarly, angiogenic switch is involved in the outgrowth of melanoma and lung carcinoma in the brain and is linked to the upregulation of vascular endothelial growth factor A (VEGF-A) [8]. In metastatic breast cancer, the angiogenic switch driven by periostin, TNC, versican, and fibronectin was associated with the sprouting of new vessels and activation of proliferative signals [10].

Here, we developed novel in vivo systems of metastatic melanomas to study mechanisms utilized by metastatic melanoma cells to persist and outgrow at distant sites including the brain. We identified FMOD, an aberrantly produced and secreted ECM molecule and SOX2, a transcription factor involved in self-renewal, as likely players that might cooperate or regulate closely coordinated programs driving the outgrowth of melanoma brain metastasis. We focused our models on brain metastasis, as this is one of the most common sites of metastatic dissemination and remains one of the primary causes of death of melanoma patients [11]. We studied these two factors via genetic approaches and through integrating studies of murine melanoma specimens with analysis of unique patient samples to establish their specific roles in the establishment of overt, clinically relevant metastases in melanoma. Our studies shed light on the dynamics of maintenance and propagation of this process and may provide new therapeutic opportunities to either prevent or treat distant metastasis in melanoma.

## Materials and methods

### Cell lines

YUGEN8, a BRAF-mutated (V600E) and PTEN-null, was established from a patient with brain metastasis (BrM) treated at Yale University. Tumor was collected with the approval of the Yale University Institutional Review Board and banked by the Yale SPORE facility. The established cell line was genotyped by Sanger sequencing and mutations were compared to the tumor from which it was derived for authentication. Cell culture was conducted as previously described [12].

### In vivo metastasis and tumor growth assays

YUGEN8 was subjected to in vivo and in vitro selection and yielded several clonally related cell line variants of indolent (Cl.1A) or highly brain-metastatic human melanoma (Cl.2A and Cl.2B) when delivered systemically through left ventricle injection. We chose these three clones, because they maintained their initial phenotypes at reinjection and after culturing and passaging for further studies. All established cell lines express luciferase allowing for tracking of metastases by bioluminescent imaging as previously described [13]. Mouse models were developed using nude mice purchased from Taconic Biosciences and used when 6 weeks old. All animal studies were approved by Yale University Institutional Animal care and Use Committee (IACUC). Full experimental details are described in the supplementary methods.

### Transcriptome profiling

Transcriptome profiling of melanoma variants parental, Cl.1A, Cl.2A, and Cl.2B cells was performed by the Yale Genome Center Analysis using the GeneChip™ Human Gene 2.0 ST Array (Thermo) that covers over 30,000 transcripts to find differentially expressed genes. Technical details on sample quality control and data pre-processing are described in the supplementary methods.

### Lentiviral constructs and guide-RNAs

All guide-RNA constructs against FMOD and SOX2 were selected from GenScript website and cloned into the Lenti-CRISPR v2 backbone under the hU6 promoter. Full details are described in the supplementary methods.

### Cell proliferation and colony formation

Cell proliferation was determined using the CellTiter-Glo® Luminescent Cell Viability Assay (Promega) as previously described [12], under both standard (10% FBS) and starvation (2% FBS) conditions. Endothelial cell proliferation was assayed using a conditioned medium collected from Cl.2A variants and recombinant human FMOD (9840-FM-050: R&D). Cell proliferation was monitored every 24 h for 3 days. Colony formation was investigated under standard or starvation conditions. After 14 days, cells were washed with PBS and stained with 0.1% crystal violet (Sigma-Aldrich) for 30 min. The number of colonies was counted under the microscope to determine colony efficiency formation.

### Endothelial cell tube formation and vascular mimicry assays

For tube formation, we used the in vitro angiogenesis assay kit (ECM640-Millipore). Briefly, 50 µl of chilled ECMatrix™ Gel Solution was added to multiple wells of a 96-well plate and incubated at 37 °C for at least 30 min to allow polymerization. HUVECs were trypsinized and counted, and 10,000 cells in serum-free endothelial cell medium were added to each well. 10 µg/ml recombinant FMOD was added to the treatment wells and 5 µg/ml recombinant VEGF was used as a positive control. For vascular mimicry, chilled 75 µl Matrigel (Corning) was added to a 48-well plate, and incubated at 37 °C for at least 30 min to allow polymerization. Cl.2A variant cells (Control, SOX2^−/−^, FMOD^−/−^, and SOX2&FMOD^−/−^) were trypsinized, counted, and 30,000 cells seeded in each well in 2% FBS cell culture medium. For both assays, HUVECs’ tube formation and vascular mimicry were evaluated by microscopy after 6 h. Multiple images per experimental group were taken and analyzed by Image J.

### Blood–brain barrier (BBB) assay

For assessing BBB leakiness, we established an in vitro BBB model as previously described [14] and based on earlier models [15, 16]. Briefly, human E6/E7/hTERT immortalized astrocytes (gift from Drs. Ranjit Bindra and Timothy Chan) and primary endothelial cells were co-cultured on opposite sides of a porous membrane in a tissue culture insert using the BD Falcon 24-Multiwell Insert System. Human astrocytes were seeded on the underside of a polylysine-treated Transwell insert (pore size of 3 µm) to create a continuous uniform monolayer. Endothelial cells were co-seeded on top of the gelatin-coated inserts, maintained for 3 days to create a compact barrier impermeable to albumin and expressing specific BBB markers. The Transwell inserts containing astrocytes and endothelial cells were gently transferred into transwells containing Cl.2A cells (Control and FMOD^−/−^) and 10 µg/ml recombinant human FMOD, hereby denoted at time 0 h. The medium inside the transwells was replaced with endothelial cell medium containing 50 µg/ml Texas-Red labeled BSA. The diffusion rate of Texas-Red BSA is a measure of the permeability/leakiness of the barrier. The fluorescent intensity of the medium in the lower chamber and the trans-endothelial electrical resistance (TEER) of the barrier were quantified at 0 and 24 hours.

### Tissue microarray construction and immunofluorescent staining

Metastatic melanoma tissue microarrays (TMA) were constructed from paraffin-embedded, formalin-fixed tissue blocks obtained from the Yale University Department of Pathology Archives with associated clinical data as approved by the Yale University Institutional Review Board. Each block was examined by a pathologist who identified a representative region of the invasive tumor for coring. Patient tumor characteristics and clinical information for both cohorts have been previously described [14, 17]. The matched cerebral-extracranial melanoma array consisted of samples from 37 patients, 32% females and 68% males. Mean age at diagnosis was 51 years (range 19–78 years) [17]. The second cohort included 169 patients, 37% females and 63% males. Mean age at diagnosis was 55 years (range 20 to 82 years) [14]. Survival time was calculated as the time from first distant metastasis diagnosis to death or last follow-up and from first melanoma BrM diagnosis to death or last follow-up. Brain metastasis-free survival was defined as time of stage IV diagnosis until brain metastasis diagnosis. The edema-to-tumor volume measurements’ volume ratio by 3D modeling from patient MRIs was derived as previously described [18]. Technical details on staining and data analysis are described in the supplementary methods.

### Statistics

We used GraphPad Prism (v 9.2.0) for statistics. For comparisons involving two experimental groups, we used an unpaired two-tailed Student’s t test. In experiments involving cell lines, three independent replicates were used as the sample size. For multiple comparisons, we applied analysis of variance (ANOVA) for significance analysis. A p value of less than 0.05 was considered significant. All quantitative data are presented as mean ± SD.

### Study ethical approval

All animal studies were approved by Yale University Institutional Animal care and Use Committee (IACUC) (Study # 2020-20152). Melanoma tumor(s) were collected with the approval of the Yale University Human Investigations Committee (Institutional Review Board, Yale HIC# 0609001869) and banked by the Yale SPORE facility.

## Results

### Development of human melanoma variants with low and high metastatic potential

**Fig. 1:**
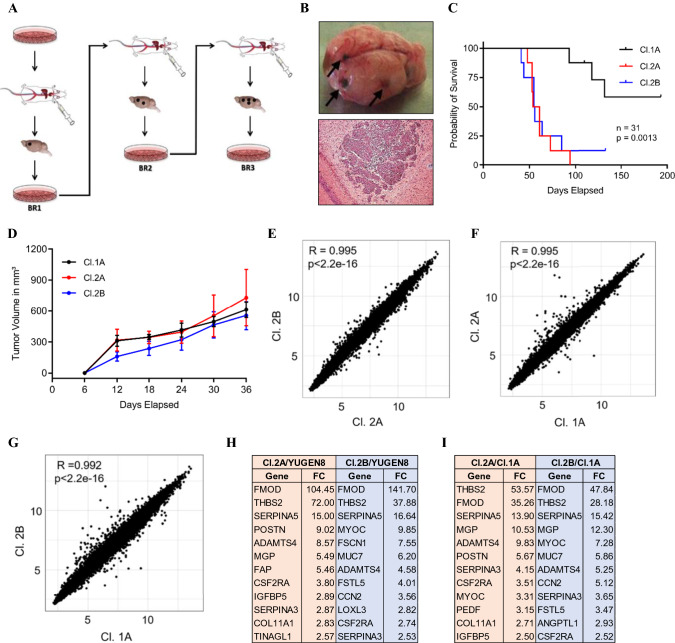
Development and genetic analysis of a highly brain-metastatic melanoma cell line model. **A** General workflow for the generation and enrichment of highly metastatic melanoma cell line clonal variants 2A and 2B from parental cell line YUGEN8. **B** Highly pigmented brain sample isolated from mice injected with Cl.2A tumor cells later confirmed by H&E staining. **C** Kaplan–Meier survival of nude mice following left ventricle injection of Cl.2A, Cl.2B, and Cl.1A cells (*p* < 0.0013, *n* = 31). **D** Tumor growth rates of Cl.1A, Cl.2A, and Cl.2B cells were implanted subcutaneously in athymic nude mice and monitored for 5 weeks (*p* > 0.05 and *n* = 18 for the three groups). Tumor growth was measured at study endpoint and tumor volumes shown were calculated using the ellipsoid volume formula. Scatter plots showing the correlation between the gene expression profiles of Cl.2A vs Cl.2B (**E**), Cl.1A vs Cl.2A (**F**), and Cl.1A vs Cl.2B (**G**), respectively. Fold changes of genes that encode for different ECM proteins overexpressed in both Cl.2A and Cl.2B vs parental YUGEN8 (**H**) and the metastatic indolent Cl.1A (**I**), respectively

To study factors regulating melanoma metastatic outgrowth, we developed animal models that recapitulate relevant steps of metastasis, including tumor cell transit through blood vessels, extravasation, and establishment of micrometastases. We reasoned that cells endowed with different metastatic potential can be isolated from the heterogeneous tumor population of melanoma metastases and can be enriched by rounds of stress and adaptation. Given the particular propensity of melanoma to metastasize to the brain, we started with brain metastasis (BrM) models. YUGEN8, a BRAF-mutated (V600E) and PTEN-null, short-term culture, established from a patient with BrM was subjected to in vivo and in vitro selection, and yielded several clonally related melanoma cell line variants with low or high brain-metastatic potential (Fig. 1A). When delivered systemically to athymic nude mice, via left ventricle (LV) injection, parental cells have a relatively low metastatic potential and induce BrM in < 50% of mice within 3 months. BrMs isolated from mice were dissociated into single-cell suspensions, expanded in culture, and subjected to two additional cycles of LV injections. We independently selected several single-cell-derived clones from cultures of successive BrM, and performed an in vivo screen for their potential to generate either slow- or fast-growing BrM when re-delivered systemically. For further studies, we chose three stable clones (Cl.1A, Cl.2A, and Cl.2B), which maintained their initial phenotypes at reinjection. All established cell lines express luciferase allowing for bioluminescence imaging (BLI) and tracking of metastases. Cl.1A is a cell line variant with indolent metastatic potential; 70% of mice injected with Cl.1A remain BrM-free at least 6 months post-injection, but disseminated melanoma cells can be recovered in culture from dissociated brain tissues. At the other extreme, Cl.2A and Cl.2B are aggressive cell line derivatives from different BrM and are endowed with significantly enhanced and preferential brain-metastatic competency; these clones induce BrM in 100% of mice as early as 3 weeks, with mice demonstrating overt brain disease by 8 weeks post-injection. The presence of macrometastases was confirmed by gross and microscopic examination (Fig. 1B). As expected, the survival of mice inoculated with indolent Cl.1A cells was significantly longer than that of mice inoculated with the aggressive Cl.2A and Cl.2B cells (Fig. 1C). Interestingly, slow-growing and aggressive brain-metastatic clones had similar subcutaneous tumorigenic potential, with no differences in mean tumor volume or survival (Fig. 1D and data not shown). To identify key molecular determinants of brain-metastatic outgrowth in our model, we conducted a transcriptomic comparison of melanoma variants with indolent or high metastatic potential. Gene expression profiles generated by these clonally related cell variants were highly correlated, indicating that modest differences emerged in their transcriptome (Fig. 1E–G). We found 50 and 61 genes (FC ± 2.5) differentially expressed between Cl.2A and Cl.1A or parental YUGEN8, respectively; 88 and 91 genes (FC ± 2.5) were differentially expressed between Cl.2B and Cl.1A or YUGEN8, respectively (Supplementary Table 1). Of these, 32 and 41 genes were common between Cl.2A and Cl.2B, when compared to Cl.1A and YUGEN8, respectively. Several genes that encode extracellular matrix (ECM) proteins and molecules that regulate their assembly were significantly enriched in the highly metastatic variants compared to indolent cells (Fig. 1H, I). The topmost differentially expressed gene in Cl.2A and Cl.2B cells compared to parental cells or Cl.1A cells was fibromodulin (FMOD).

### FMOD is a mediator of melanoma metastatic progression

**Fig. 2:**
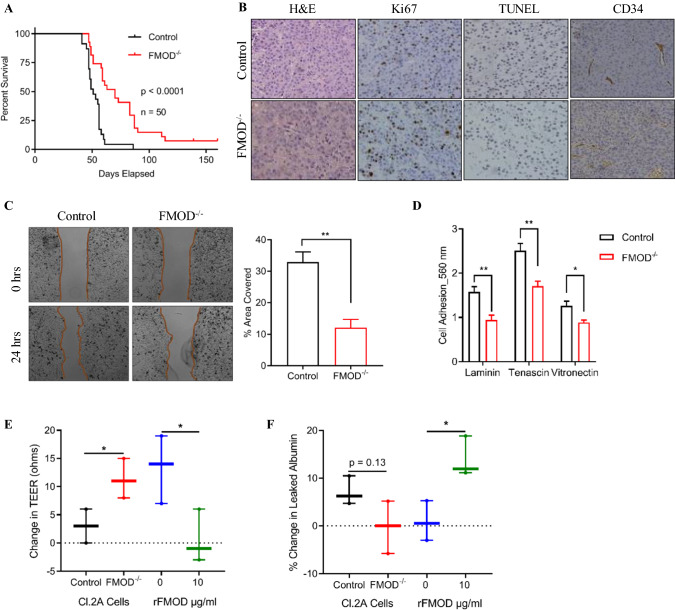
In vivo and in vitro effects of FMOD silencing in Cl.2A cells. **A** Kaplan–Meier curves depicting survival of athymic mice following left ventricle injection with Cl.2A cells (Control vs FMOD^−/−^; *p* < 0.0001, *n* = 50). **B** Immunohistochemical staining showing the expression of Ki67, TUNEL assay, and expression of CD34 in tumors isolated from mice in Cl.2A Control vs FMOD^−/−^ (Original magnification: × 10). **C** FMOD depletion inhibits Cl.2A cell migration as demonstrated by gap closure assay and **D** adhesion to laminin, tenascin, and vitronectin as shown by cell adhesion assay. **E** Changes in TEER in an in vitro blood–brain barrier model show high TEER value in the presence of FMOD^−/−^ cells, a phenotype reversed by rFMOD treatment. **F** High TEER value in FMOD^−/−^ as shown in Fig. 2E is associated with low levels of leaked albumin. The opposite is seen in the presence of rFMOD. Data are expressed as mean ± SD of three biological replicates and statistical significance is determined using a two-sided Student’s t test:*p < 0.05, **p < 0.01, ***p < 0.001, and ****p < 0.0001

Early and overt brain disease was only achieved by Cl.2A and Cl.2B cells, which retain production and secrete high levels of FMOD in culture, suggesting that its dysregulation might be directly linked to achieving high brain-metastatic competence (Supplementary Fig. 1A). Utilizing the CRISPR/Cas9 technique, we successfully silenced FMOD in Cl.2A cells, and thereafter, we investigated the biological consequences of FMOD loss (Supplementary Fig. 1B-C). FMOD depletion decreased the BrM activity of Cl.2A cells, which was evident by BLI, and we observed a statistically significant difference in survival between these mice and the control group (Fig. 2A). In addition, the incidence of brain macrometastasis was significantly lower (60%) with FMOD silencing compared to control (Supplementary Table 2). FMOD depletion slightly attenuated extracranial metastasis (Supplementary Table 2), though the difference was not statistically significant. Brain tumors from both control and FMOD-depleted variant cells showed similar staining for the proliferation marker Ki67 and TUNEL reaction for apoptosis, as well as for the endothelial cell-specific marker CD34 (Fig. 2B and Supplementary Fig. 1D, E).

To understand the mechanism by which FMOD regulates brain-metastatic outgrowth in our model, we investigated its role in several rate-limiting steps of the metastatic cascade. Once in the arterial circulation, cancer cells can extravasate, survive, and then arrest or develop as perivascular lesions. We first evaluated the in vitro tumorigenic potential of Cl.2A cells. FMOD silencing did not affect cell proliferation either under standard culture or starvation conditions (Supplementary Fig. 1F). Assessment of annexin V/propidium iodide staining showed that suppressing FMOD does not induce spontaneous cell death in our model, and hence, no effects on cell viability were noted (Supplementary Fig. 1G). Likewise, FMOD depletion did not impair colony formation whether cultured under standard conditions or serum starvation (Supplementary Fig. 1H). Next, we investigated the role of FMOD in cell motility in vitro*,* using the gap closure assay. FMOD knockout in Cl.2A cells significantly attenuated cell migration (Fig. 2C), but did not affect invasion (Supplementary Fig. 1I). To form BrMs, cells need to transmigrate through the BBB which requires them to first adhere to the brain endothelium, rich in laminin and collagen IV [19]. FMOD silencing significantly decreased the adhesion of Cl.2A cells to tenascin, laminin, and vitronectin (Fig. 2D) but not fibronectin, collagen I, II, or IV. Permeability of tumor vasculature is altered in advanced disease. To test the effect of FMOD on the BBB, we assessed tight junction integrity by measurement of trans-endothelial electrical resistance (TEER) and albumin leakiness in an in vitro co-culture model comprised of human astrocytes and primary human endothelial cells (HUVECs), which we previously developed [14]. We showed that HUVECs, though are extracranial endothelial cells, have the ability to acquire brain endothelial cell characteristics and tight junctions [14]. We observed a significant increase in TEER in the presence of FMOD-depleted cells compared to controls, a phenomenon that was reversed in the presence of recombinant FMOD (Fig. 2E). The increase in BBB permeability in the presence of FMOD was confirmed by analysis of Texas-Red albumin extravasation through transwells (Fig. 2F). This suggests that FMOD might promote melanoma brain-metastatic relapse by altering endothelial barrier functions to facilitate the extravasation of cancer cells into the brain parenchyma.

**Fig. 3:**
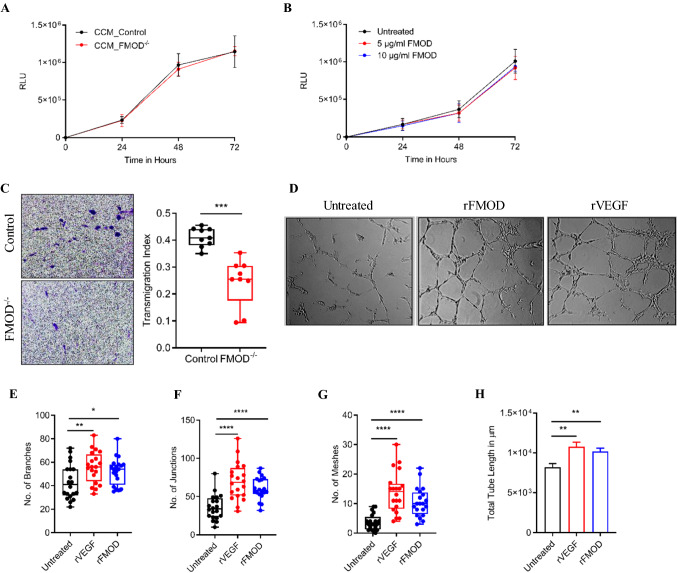
In vitro role of FMOD in tumor angiogenesis. Sub-culture of human endothelial cells (HUVECs) with either conditioned medium from Cl.2A cells (Control vs FMOD−/−) (**A**) or recombinant human FMOD (**B**) has no effect on HUVECs proliferation. **C** Transmigration potential of HUVECs across a fibronectin-coated barrier toward Cl.2A cells is significantly reduced upon the loss of FMOD. **D** Representative images of HUVECs tube formation following treatment with either recombinant human FMOD or VEGF (Scale 100 µm). Quantification and analysis of the number of branches (**E**), junctions (**F**), meshes (**G**), and the total tube length (**H**) of HUVECs following treatment with either recombinant FMOD or VEGF. All data are expressed as mean ± SD of three biological replicates and statistical significance is determined using a two-sided Student’s *t* test: *p < 0.05, **p < 0.01, ***p < 0.001, and ****p < 0.0001

Previous studies demonstrated that metastatic cells entice local endothelium to form angiogenic vessels [20]. FMOD was previously linked to angiogenesis in several malignancies [21, 22]. To explore the role of FMOD in angiogenesis at the cellular level, we studied the effects of Cl.2A cells, in a co-culture system, or recombinant FMOD on endothelial cells. We found no differences in the proliferation of HUVECs when sub-cultured in a conditioned medium from Cl.2A cell variants or in the presence of recombinant FMOD. (Fig. 3A, B). Endothelial cell transmigration is an important step during angiogenesis regulated by chemotactic stimuli and involves the degradation of ECM to allow passage of the migrating cells [23]. HUVECs’ transmigration rates toward Cl.2A FMOD-depleted cells in an indirect co-culture Transwell system were significantly slower than toward control cells (Fig. 3C). Finally, we evaluated HUVEC’s ability to form tube-like structure (TLS) in the presence of recombinant human FMOD. Consistent with previous reports [21, 22], FMOD treatment-induced HUVEC tube formation and its effects were comparable to those induced by vascular endothelial growth factor (VEGF), a potent angiogenic molecule (Fig. 3D). We noted an increased number of branches, junctions, meshes, and total tube length in treated compared to non-treated cells (Fig. 3E–H). These findings suggest that the metastatic aggressiveness of Cl.2A cells may result from FMOD’s promotion of angiogenesis.

### SOX2 is a rate-limiting step of metastatic outgrowth upon FMOD silencing

**Fig. 4:**
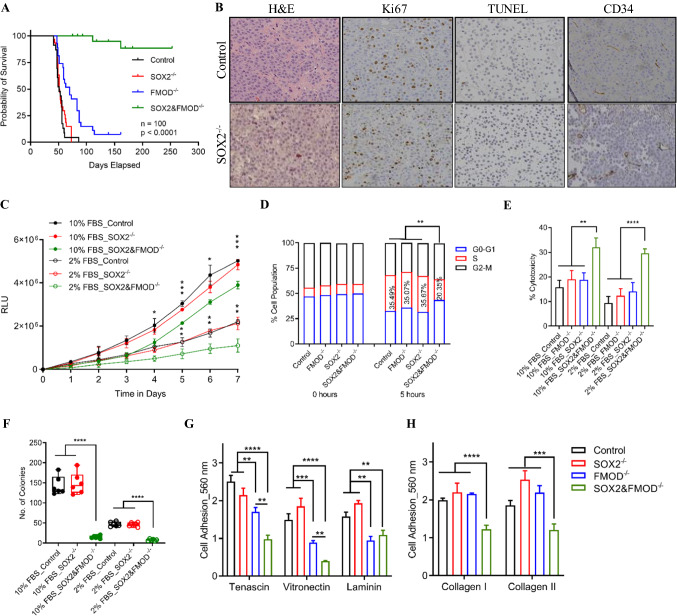
In vivo and in vitro effects of dual FMOD and SOX2 knockout in Cl.2A cells. **A** Kaplan–Meier survival curves of athymic nude mice following left ventricle injection with Cl.2A cells (Control vs FMOD^−/−^ vs SOX2^−/−^ vs SOX2&FMOD^−/−^; *p* < 0.0001, *n* = 105). **B** Immunohistochemical staining showing the expression of Ki67, TUNEL assay and expression of CD34 in tumors isolated from mice in Cl.2A Control vs SOX2^−/−^ (original magnification:  x10). Representative images from SOX2&FMOD^−/−^ are missing as mice in this group barely formed any tumors (2/25). **C** Depletion of SOX&FMOD attenuated cell proliferation under both standard culture conditions and starvation. **D** The decreased cell proliferation in SOX&FMOD−/− is associated with a slow cell-cycle transition from G1 to S as depicted by the low percentage of these cells in the S phase. **I** MTT assessment of cytotoxicity in Cl.2A cells after treatment with etoposide for 48 hours. Loss of both SOX2 and FMOD significantly impacts colony formation in Cl.2A cells (**F**), reduces the cell adhesion capacity of Cl.2A cells to tenascin and vitronectin compared to FMOD-depleted cells (**G**), and impacts Cl.2A cells adhesion to both collagen I and II (**H**). All data are expressed as mean ± SD of three biological replicates and statistical significance is determined using a two-sided Student's *t* test: *p < 0.05, **p < 0.01, ***p < 0.001, and ****p < 0.0001

Though FMOD depletion significantly inhibited BrM, we hypothesized that FMOD is not functioning independently to drive the expansion of tumor foci into macrometastases. We elected to study the SOX2 transcription factor, a critical regulator of self-renewal, because, like FMOD, its protein expression was elevated in the aggressive Cl.2A and Cl.2B cells and lost in the metastatic impaired Cl.1A variant (Supplementary Fig. 1A). SOX2 expression significantly and selectively promotes metastasis to the brain in breast cancer [19]. We therefore examined if SOX2 was required for or contributed to metastatic formation at distant sites in our model. We successfully ablated the expression of SOX2 or SOX2 and FMOD in Cl.2A cells using CRISPR/Cas9 (Supplementary Fig. 1B, C). For reproducibility, we carried out two independent transductions from which stable clones were selected and studied further. Silencing SOX2 did not affect metastasis as we observed similar rapid weight loss and overt brain-metastatic colonization in 100% of mice following systemic injection with either SOX2-depleted or control Cl.2A cells. By contrast, 92% of mice injected with SOX2 and FMOD-depleted Cl.2A cells failed to develop BrM even after 6 months of follow-up (Supplementary Fig. 2A). Moreover, macroscopic metastases at sites other than the brain remained undetectable for over 6 months in 76% of mice, as indicated by the absence of bioluminescent signal (Supplementary Fig. 2A and Supplementary Table 2). The marked inhibition of metastatic outgrowth was confirmed by a statistically significant difference in the survival rates between the experimental and control groups (Fig. 4A). The impaired metastatic capacity of dual SOX2 and FMOD-depleted cells was confirmed by microscopic examination of H&E staining of histological brain sections at the experimental endpoint. Pathology revealed rare, small tumor foci that did not develop into macroscopic metastasis (Supplementary Fig. 2B). By contrast, the brains of mice inoculated with control or SOX2-depleted cells showed multiple large lesions (Fig. 4B). Brain tumors derived from either SOX2-depleted or control cells showed similar Ki67, TUNEL and CD34 staining (Fig. 4B and Supplementary Fig. 2C). SOX2 expression is binary, while its accumulation is a dynamic process; tumor cells accrue varying levels of SOX2 as they actively proliferate [24–26]. To verify if SOX2 was present only in a distinct subpopulation of cells, we conducted immunofluorescence staining and found SOX2 positive staining in the majority of DAPI-positive Cl.2A cells (Supplementary Fig. 2D). These results suggested that FMOD-regulated tumor–host interactions might be coupled with SOX2-driven signaling mechanisms in BrM in our model.

We next examined concomitantly the functions of SOX2 and FMOD. Similar to FMOD, depleting SOX2 did not alter the proliferation rates of Cl.2A cells. In contrast, dual silencing of SOX2 and FMOD inhibited cell proliferation under both standard culture conditions and serum starvation, suggesting that FMOD expressing Cl.2A cells are more capable of overcoming stress conditions (Fig. 4C). The reduction in proliferation was likely attributable to cell-cycle modulation. Flow cytometry analysis showed that dual silencing of SOX2 and FMOD inhibited cell-cycle progression via delayed G1 to S phase transition (Fig. 4D). Neither SOX2 depletion nor SOX2 and FMOD dual silencing had an effect on spontaneous apoptosis (Supplementary Fig. 2E). However, in the presence of etoposide, a topoisomerase II inhibitor, which induces apoptosis, SOX2 and FMOD dual silencing caused elevated levels of cytotoxicity under both, standard conditions and serum starvation (Fig. 4E). The ability of cells to form colonies reflects their capacity to initiate tumor growth. Dual SOX2 and FMOD depletion impeded colony formation in Cl.2A cells (Fig. 4F). To explore the relevance of these findings in vivo, we compared subcutaneous tumor growth rates of Cl.2A variants and found no significant differences (Supplementary Fig. 2F). Lowering the number of transplanted cells from 300,000 to 3000 per flank did not affect tumor formation (data not shown). This suggests that the indolent metastatic phenotype generated by SOX2 and FMOD dual knockout in the aggressive clone is independent of the intrinsic capacity of Cl.2A cells to proliferate and possibly controlled by specific microenvironmental factors in metastatic sites.

We next assessed the migratory and invasive properties of SOX2 depleted cells. SOX2 silencing did not affect cell motility in a wound-healing assay. SOX2 and FMOD dual gene editing had no additive inhibitory effects on cell migration when compared to FMOD depletion (Supplementary Fig. 2G). There were no significant differences between the matrigel invasion indices of Cl.2A variant cells when compared to control cells (Supplementary Fig. 2H). As we previously found that FMOD depletion significantly decreased the adhesion of Cl.2A cells to tenascin, laminin, and vitronectin, we continued this study with regard to SOX2 depletion. SOX2 silencing did not alter the adhesion of Cl.2A cells to any of the ECM components tested. However, dual SOX2 and FMOD inhibition further decreased Cl.2A adhesion to tenascin and vitronectin compared to FMOD depletion only, with no additive effect on laminin (Fig. 4G). Interestingly, SOX2 and FMOD dual gene editing significantly impaired cell adhesion to collagen I and II, indicating the presence of a fine-tuned cooperative mechanism between SOX2 and FMOD cellular programs (Fig. 4H). Notably, SOX2 silencing had no effect on endothelial cell proliferation and did not significantly alter the effects of FMOD depletion on HUVECs transmigration in vitro (Supplementary Fig. 2I, J). Our data suggest that SOX2 and FMOD might have overlapping and distinct functions.

**Fig. 5:**
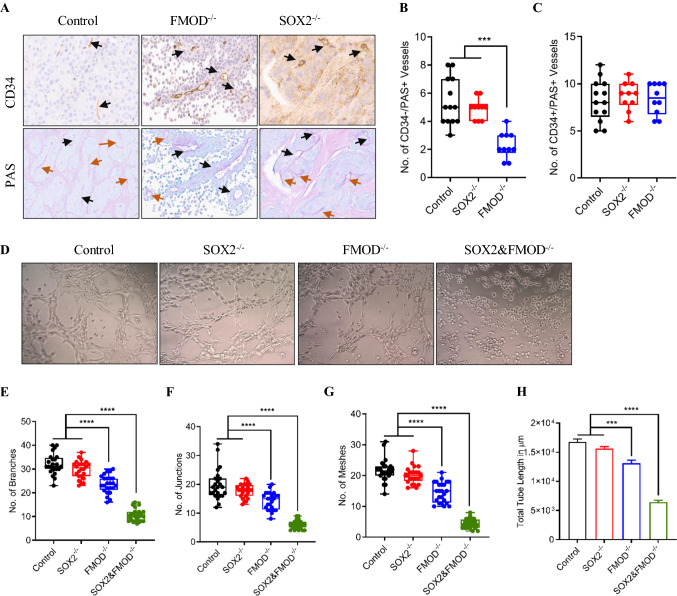
In vivo and in vitro effects of dual FMOD and SOX2 knockout in vascular mimicry. **A** Representative images of immunohistochemical staining for CD34 (angiogenesis) and PAS (vascular mimicry) in control, FMOD^−/−^, and SOX2^−/−^ tumors isolated from mice brains (black arrow: CD34+/PAS+, brown arrow: PAS+, original magnification:  x10) Representative images from SOX2&FMOD^−/−^ are missing as mice in this group barely formed any tumors (2/25). **B** Analysis of PAS+/CD34− vessels in these tumors showed decreased vascular mimicry in brain metastases from the FMOD^−/−^ group. **C** The number of PAS+/CD34 + microvessels and vessels in the three tumor groups were comparable. Data analysis included at least two different regions per tumor per mouse. **D** Representative images (scale 100 µm) showing suppression of vascular mimicry formation of Cl.2A cells in vitro upon dual FMOD and SOX2 silencing. Quantification and analysis of the number of branches (**E**), junctions (**F**), meshes (**G**), and the total tube length (**H**) of Cl.2A cells. All data are expressed as mean ± SD of three biological replicates and statistical significance is determined using a two-sided Student’s *t* test: *p < 0.05, **p < 0.01, ***p < 0.001, and ****p < 0.0001

Upon examination of the H&E stained tumor sections formed by Cl.2A, we noted the presence of vessel-like structures seemingly formed by tumor cells. Like other type of cancer cells, melanoma cells form vascular-like structures, a process recognized as a form of neovascularization, independent of angiogenesis, and referred to as vasculogenic mimicry (VM) [27, 28]. To determine whether VM was present in BrM collected from our models, we conducted thorough microscopic examination of sections for CD34 immunohistochemical and Periodic acid-Schiff (PAS) histochemical staining. A specific feature of VM channels is a wall structure negative for CD34 staining but positive for PAS staining [28, 29]. Quantification of PAS( +)/CD34(-) vessels in tumor xenografts showed a reduction in VM content in tumors with FMOD depletion compared to tumors formed by Cl.2A cells or SOX2 knockout cells (Fig. 5A, B). However, the number of PAS(+)/CD34(+) vessels was comparable among the three tumor groups. (Fig. 5C). Since dual SOX2 and FMOD impeded the formation of macrometastasis, it was impossible to assess VM in this group. Next, we sought to verify the formation of tubal structures derived from our cells in vitro. Melanoma cells cultured on matrigel readily form tubular-like structures in vitro [30]. As expected, Cl.2A cells exhibited VM in a tube formation assay conducted. Consistent with our findings in tumor xenografts, FMOD depletion partially reduced VM in vitro (Fig. 5D), as noted by the decreased number of branches, junctions, meshes, and total tube length compared to control cells (Fig. 5E–H). SOX2 depletion did not affect VM. Interestingly, cells inhibited for both FMOD and SOX2 expression completely lost the ability to form tube-like structures, suggesting once again a possible cooperation between SOX2 and FMOD-driven cellular programs (Fig. 5D–H).

### Gene signatures of FMOD- and/or SOX2-silenced cells

**Fig. 6:**
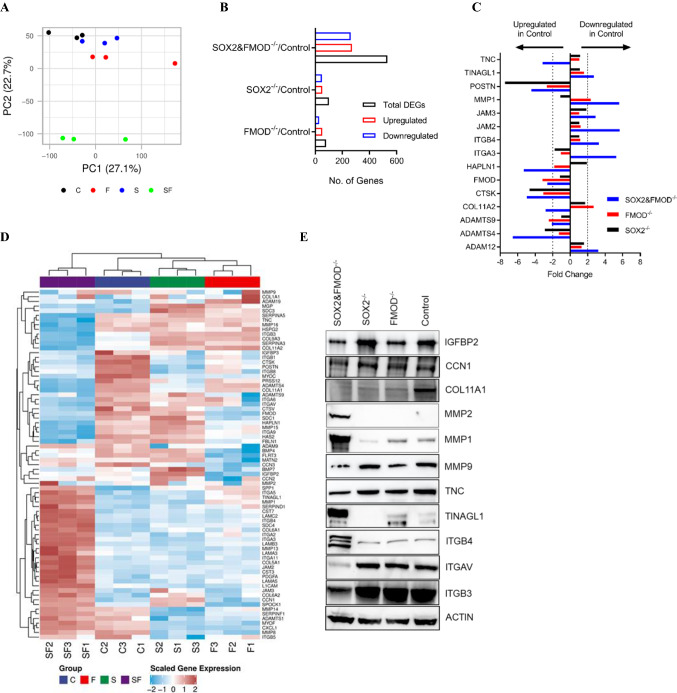
Transcriptomic profiling of Cl.2A cell variants. **A** Principal component analysis (PCA) demonstrates the relationship between the different variants of Cl.2A cells; SOX2&FMOD^−/−^ group clusters separately and is therefore distinct from the rest (C: Control, F: FMOD^−/−^, S: SOX2^−/−^, and SF: SOX2&FMOD^−/−^). **B** Bar graph showing the number of differentially regulated genes in Cl.2A variants when compared to control group. **C** Mean fold changes of different ECM genes, either overexpressed or under-expressed, in the control group compared to SOX2^−/−^, FMOD^−/−^, and SOX2/FMOD^−/−^. **D** Heatmap comparison of the ECM signature in the four groups reveals a distinct fingerprint in the SOX2/FMOD^−/−^ group. **E** Immunoblots showing the protein expression of different ECM proteins and/or regulators of the ECM in Cl. 2A cells upon silencing of SOX2, FMOD, or both SOX2 and FMOD

To gain insights into how distant metastasis is regulated in our model, changes in the gene expression profiles of Cl.2A cells after suppression of FMOD and/or SOX2 were determined by microarray analysis. We focused primarily on changes following single FMOD or double FMOD/SOX2 silencing as these genetic approaches induced significant differences in vitro and in vivo*.* By hierarchical clustering and principal component analysis, we found that FMOD/SOX2 double knockout samples cluster separately from other samples, indicating that these cell populations are biologically distinct, and supporting their dramatic phenotypic differences (Fig. 6A). By comparative analyses and using the FC ± 2.0 cut-off, we identified 80 differentially expressed genes (51 upregulated and 29 downregulated) in the FMOD-silenced cells and 531 differentially regulated genes (271 upregulated and 260 downregulated) in the SOX2/FMOD-silenced cells (Fig. 6B and Supplementary Table 3). To classify the differentially expressed genes into biological categories, gene sets were further subjected to enrichment analysis using the NCBI human genome as a reference list. Enriched functional categories included transcription misregulation in cancer, cell cycle, ECM–receptor interaction, focal adhesion, PI3K-AKT/TNF/IL-17/JAK-STAT signaling pathways, and cytokine–cytokine receptor interaction (Supplementary Table 4). Loss of FMOD expression was concomitantly associated with the dysregulated expression (FC ± 2.0) of 12 ECM-remodeling genes including *POSTN*, *MMP1*, *COL11A2*, *CTSK*, *LIF*, and *SPP1* (Supplementary Table 5). Dual SOX2/FMOD silencing led to the increased number of dysregulated ECM-remodeling genes (FC ± 2.0) including *POSTN*, *COL11A1*, *COL11A2*, *ADAMTS4*, *HAPLN1*, *TNC*, *TINAGL1*, *ITGB4*, *SPP1*, *MMP1*, and *MMP9*, among others (Fig. 6C–E and Supplementary Table 5). We concluded that modulation of these ECM-remodeling proteins likely interferes with tumor–matrix crosstalk and is at least in part responsible for the dramatic difference in the metastatic phenotype of our models.

### Effects of FMOD and/or SOX2 on Hippo pathway signaling

**Fig. 7:**
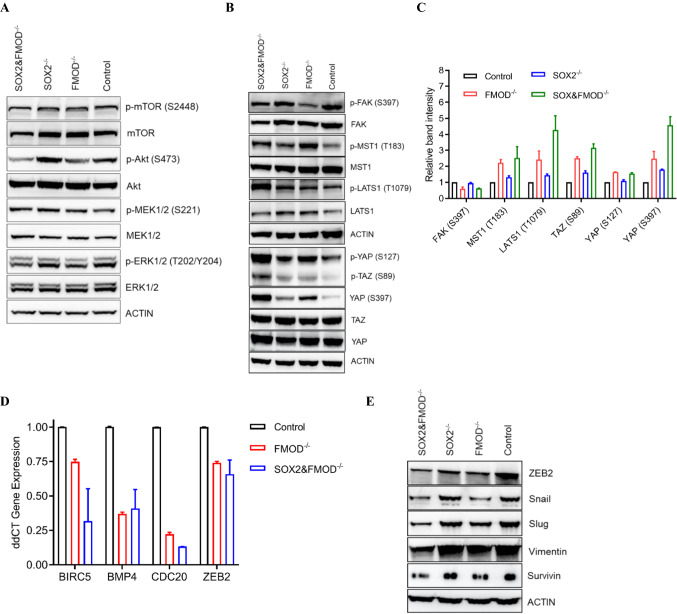
FMOD and dual FMOD/SOX2 knockouts activate the Hippo pathway. **A** Immunoblot illustration of key members of the MAPK and PI3K-Akt pathways. **B** Immunoblot showing the expression of FAK (S397) and phosphorylation levels of members of the Hippo pathway (MST1/2, LATS1, and YAP/TAZ) in Cl.2A control and FMOD- and/or SOX2-knock-out cells. **C** Immunoblot band intensity analysis of the phospho-proteins shown in Fig. 7B from three biological replicates. **D** Quantitative PCR of gene expression levels of different YAP/TAZ targets, that have been implicated in melanoma upon FMOD or FMOD and SOX2 depletion **E** Immunoblot of several EMT factors (downstream of YAP/TAZ) involved in vascular mimicry in Cl.2A cells showing decreased expression in both FMOD^−/−^ and SOX2&FMOD^−/−^ groups

We next sought to uncover the signaling mechanisms tied to the aforementioned phenotypic alterations. Since our models are derived from a BRAF^V600E^ PTEN-null melanoma, we first evaluated changes in PI3K/Akt and MAPK signaling associated FMOD and/or SOX2 loss. Though FMOD-depleted Cl.2A cells showed a slight decrease in p-Akt (S473) levels compared to control, this was not associated with changes in p-mTOR (S2448) or downstream effectors p-4EBP1 (S65) and p-RPS6 (S235/236) (Fig. 7A and data not shown). Furthermore, loss of FMOD resulted in negligible changes in p-ERK1/2 (T202/Y204) levels and no changes in p-MEK1/2 (S221) (Fig. 7A). FMOD is a ubiquitous component of the ECM involved in structural matrix assembly and organization [6]. Since the Hippo pathway has been linked to ECM organization and remodeling [31], we explored the possible effects of FMOD on Hippo signaling. Loss of FMOD was associated with decreased p-FAK (S397) and activation of the Hippo pathway via phosphorylation of its upstream mediators, MST1 (T183) and LATS1 (T1079) (Fig. 7B, C). Downstream of LATS1, we observed increased p-YAP (S127 and S397) and p-TAZ (S89) in both FMOD- and SOX2/FMOD-depleted cells (Fig. 7B, C). To confirm the modulation of YAP/TAZ activity, we quantified mRNA expression levels of some of the known YAP/TAZ target genes, including *BMP4*, *BIRC5*, *CDC20*, and *ZEB2* [32]. We found decreased expression of these genes in both FMOD- and SOX2/FMOD-depleted cells, (Fig. 7D). Interestingly, loss of FMOD and SOX2/FMOD reduced the expression of several known YAP/TAZ targets implicated in vascular mimicry, including ZEB2, SNAIL, and SLUG, which are all key transcription factors that regulate the epithelial–mesenchymal transition (EMT) process, the mesenchymal markers vimentin, and survivin, also implicated in EMT (Fig. 7E) [33]. Collectively, these data indicate that FMOD and SOX2 might antagonize with Hippo signaling to regulate YAP/TAZ transcriptional activity.

### Studies of FMOD and SOX2 expression and their prognostic value in melanoma patient samples

**Fig. 8:**
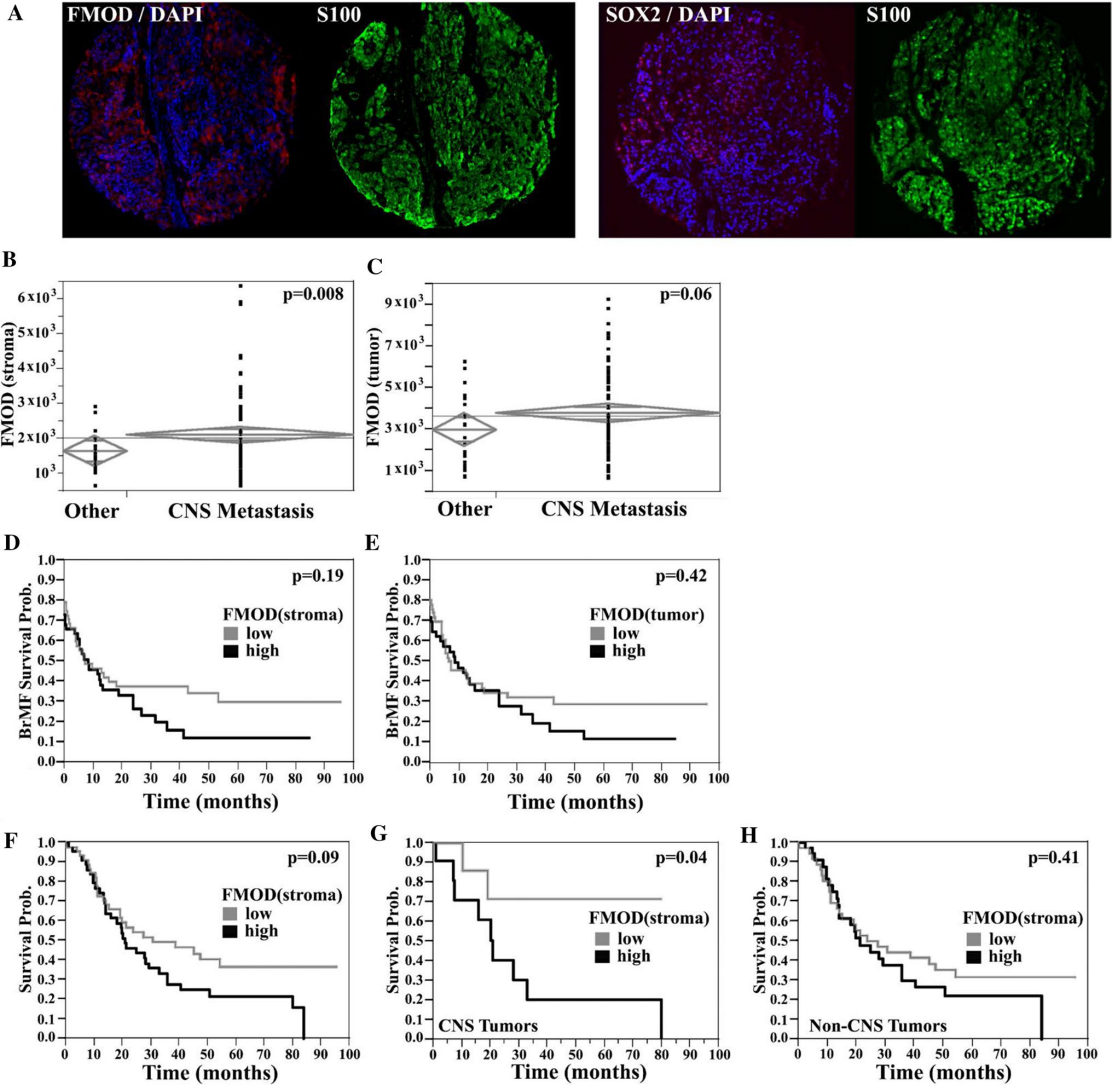
Analysis of FMOD expression in melanoma patient samples. **A** Representative quantitative Immunofluorescence staining (QIF) of FMOD or SOX2 in a BrM melanoma case. Two different staining patterns are being shown: for FMOD, predominantly stromal and for SOX2, predominantly nuclear. Unpaired *t* tests, showing that expression of FMOD (continuous QIF scores) in the stromal (**B**) or tumor (**C**) compartments was higher in patients who developed brain metastases at any time during the course of illness compared to patients who did not (designated’Other’). Kaplan–Meier curves demonstrate the correlation between FMOD expression (dichotomized scores in the stroma) (**D**) or S100-positive tumor (**E**) and Brain Metastasis Free Survival (BMFS). **F** Kaplan–Meier curves demonstrate the correlation between FMOD expression (dichotomized scores in the stroma) in CNS (**G**) or extracranial (Non-CNS) (**H**) metastatic melanomas and Overall Survival (OS). High FMOD expression in cerebral specimens was significantly associated with decreased overall survival

We next studied FMOD expression in human melanoma samples employing quantitative immunofluorescence and tissue microarrays. We started with a cohort of 169 metastatic melanoma patients with variable times to development of BrM [14]. The fluorescent signal was quantified within the S100-positive area (tumor expression) or the tumor microenvironment (stromal expression) (Fig. 8A). There was a strong correlation between stroma and tumor FMOD expression (*R* = 0.78, *p* < 0.0001) (Supplementary Fig. 3A). FMOD in the stroma and tumor was higher in patients who developed BrMs at some point during their illness (*t* test, *p* = 0.008 and *p* = 0.06, respectively) (Fig. 8B, C). However, there was no correlation with the incidence of metastasis at other sites. This suggests that FMOD dysregulation might be preferentially associated with cerebral metastasis. We also evaluated matched cerebral and extra-cerebral metastases from a cohort of 37 melanoma cases previously described [17]. FMOD levels in cerebral metastases were not significantly different when compared to their extra-cerebral counterparts (data not shown), indicating that FMOD dysregulation is likely an early event in BrM. Examination of FMOD distribution across various metastatic sites showed a prevalence of higher stromal FMOD in visceral metastases and low expression in bone lesions (ANOVA, *p* = 0.03) (Supplementary Fig. 3C). We next assessed the association between FMOD levels and organ-specific metastasis-free survival. There was no association between FMOD expression and time to development of brain, liver, lung, or bone metastasis (Fig. 8D, E and data not shown). Increased stromal FMOD correlated with decreased overall survival (OS), though data only trended toward significance (log-rank *p* = 0.09) (Fig. 8F). When evaluating FMOD specifically in intracranial tumor stroma, high expression was significantly associated with decreased OS and this was confirmed in our smaller cohort of brain lesions (RR 1.99; Lower CL 1.00; Upper CL 5.10, *p* = 0.04; Fig. 8G and data not shown). No correlation was found between FMOD expression and gender, M stage, LDH levels, or BRAF/NRAS mutational status, but levels were higher in patients over 50 years old (t test, *p* = 0.02). Using anti-CD34 to identify endothelial cells, we found no association between FMOD and vascular density, a finding which was validated in our second cohort of cases (*t* test, *p* = 0.56; Supplementary Fig. 3D and data not shown). Interestingly, elevated FMOD levels were found in cranial lesions with a higher edema-to-tumor volume ratio by 3D modeling (*t* test, *p* = 0.02; Supplementary Fig. 3E) [18].

**Fig. 9:**
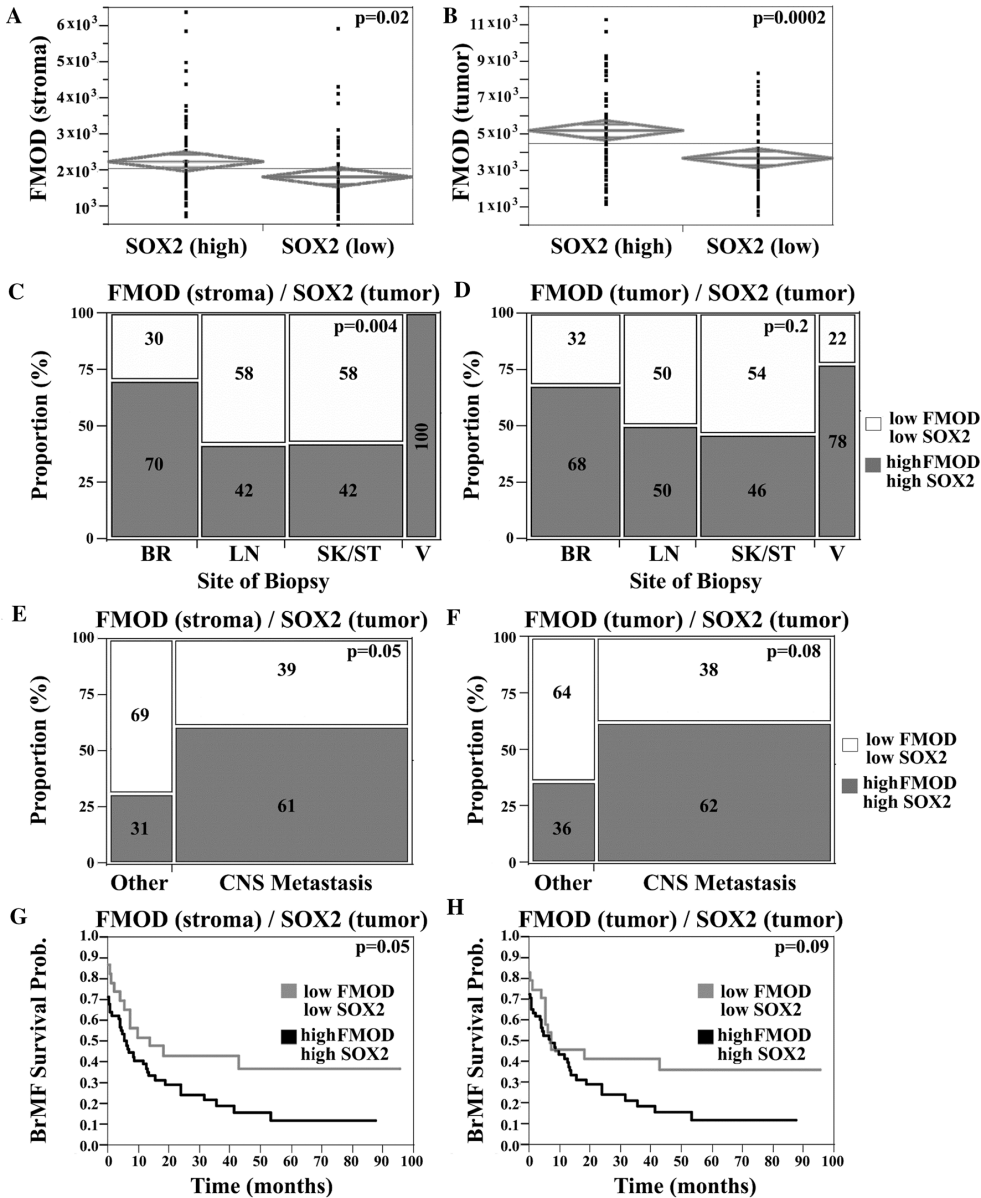
Combined analysis of FMOD and SOX2 expression in melanoma patient samples. Two sample t test, showing that high FMOD expression (continuous FMOD intensity scores) in the stromal (**A**) or tumor (**B**) compartments was significantly associated with high SOX2 tumor expression (nuclear scores dichotomized by the median value). Chi-squared (*χ*^2^) analysis was used to compare FMOD and SOX2 staining, among specimens of different origins. FMOD in the stromal (**C**) or S100-positive tumor (**D**) compartments and tumor nuclear SOX2 scores were arbitrarily dichotomized to ‘high’ and ‘low’ categories by the associated median value for each molecule. Two groups of patients were defined as follows: one group had ‘low’ FMOD and ‘low’ SOX2 expression; the second group had 'high’ FMOD and ‘high’ SOX2 expression. There was a significantly higher proportion of cases with ‘high’ levels of both FMOD (stroma) and SOX2 in BrMs relative to other sites. Chi-squared (*χ*^2^) analysis shows that the proportion of cases with both ‘high’ FMOD in the stromal (**E**) or tumor (**F**) compartments and ‘high’ SOX2 was higher in patients who developed brain metastases at any time during the course of illness compared to patients who didn’t (designated’Other’). Kaplan–Meier curves showing the association between high/low FMOD/SOX2 expression and BMFS.’High’ stromal FMOD expression and ‘high’ SOX2 expression were significantly associated with decreased BMFS (*BR* brain, *LN* lymph node, *SK* skin, *ST* soft tissue, *V* viscera)

We next sought to investigate the possible combined effect of FMOD and SOX2 upregulation, in the context of clinical outcome. SOX2 staining was heterogeneous with a minority of positive tumor cells (Fig. 8A), a pattern consistent with its roles in a distinct subpopulation of cells such as ‘stem-like’ cells. SOX2 expression did not correlate with organ-specific metastasis or survival (Supplementary Fig. 3F, G), suggesting that SOX2 dysregulation by itself is not associated with either a brain homing phenotype or with aggressive disease in general. High FMOD in either stroma or tumor was significantly associated with high SOX2 expression and this relationship held when cerebral lesions were examined separately (*t* test, *p* = 0.02 and *p* = 0.0002, respectively; Fig. 9A, B). We next generated a composite FMOD/SOX2 score. We defined two groups of patients: high FMOD/high SOX2 and low FMOD/low SOX2. We found a higher prevalence of cases with elevated levels of both FMOD and SOX2 in BrM relative to other sites (*χ*^2^-analysis, 70% of cases vs. 42%, respectively, *p* = 0.004; Fig. 9C). In addition, we found an increased frequency of tumors with high levels of both proteins in patients who developed BrMs at some point during their illness (*t* test, *p* = 0.05 and *p* = 0.08, in the stroma and tumor, respectively; Fig. 9E, F). Moreover, patients with high FMOD in the stroma and high SOX2 had a higher risk for early BrM development (RR 1.32; Lower CL 0.99; Upper CL 1.82, *p* = 0.05; Fig. 9G). A similar trend was seen for cases with high tumor FMOD and SOX2 expression (*p* = 0.09; Fig. 9H). No association was found between this composite score and metastasis at other sites or OS. Our data suggest a possible role of SOX2 in tumors with FMOD dysregulation; concomitant SOX2 upregulation might contribute to its association with the early development of melanoma BrMs, but not with disease aggressiveness.

## Discussion

We developed and characterized novel xenograft models of slow- and fast-growing human melanoma with propensity to colonize the brain. Our model is similar to an earlier model developed by other researchers in that the clonally related derivatives with different brain-metastatic phenotypes originated from the same patient-derived melanoma, passaged in the murine brain [34]. The main distinction in our model is that clones endowed with distinct metastatic competence were isolated from dissociated bulk tumor tissue and single-cell-derived. Our systems, therefore, recapitulate metastatic heterogeneity within tumor cell populations that undergo diverse reprogramming to adapt to a continuously changing microenvironment. Though our clonal variants showed similar growth rates in subcutaneous xenografts, the slow-growing Cl.1A variant was inefficient to form BrM when injected intracardially. This observation highlights the importance of the microenvironment and the plasticity of the molecular program(s) that can either stimulate or inhibit metastatic outgrowth in our models.

FMOD was the most differentially expressed gene in the aggressive variants, suggesting that it might be functionally linked to their metastatic competence. FMOD is a secreted component of the ECM, a dynamic network of macromolecules that modulates interactions between tumor cells and stroma [3, 35]. Physiologically, FMOD is a regulator of collagen fibrillogenesis, a potent angiogenic factor, and a critical component of the stem cell niche [23, 36–40]. In cancer, it has been linked to cell migration, apoptosis, and angiogenesis [35]. FMOD has yet to be linked to metastasis-initiating competence, while its roles in melanoma are currently unknown. Loss of FMOD significantly delayed BrM in our model, suggesting that its dysregulation might be specifically associated with melanoma brain colonization and growth. This was confirmed in our study of patient tumors; FMOD was upregulated in BrMs, and its expression in intracranial tumors predicted a poor overall survival.

We demonstrated that FMOD is not essential for cell growth or survival but might facilitate metastasis by enhancing cell motility by interfering with integrin-FAK signaling. This was consistent with a study in glioma, showing that FMOD regulates cell migration via activation of the integrin-FAK-dependent pathway [41]. Our expression analysis further strengthens this link as FMOD depletion correlated with altered expression of several integrins. These data support the hypothesis that mechanistically FMOD might interfere with the activation of integrins or other cell surface receptors to regulate migration and facilitate metastasis.

The angiogenic switch that activates quiescent local vasculature to form new blood vessels is a critical step in growing tumors. Our in vitro data suggests that FMOD might promote angiogenesis via inducing endothelial cell migration, sprouting, and capillary formation. This is in line with several studies that showed FMOD is a critical regulator of angiogenesis [21, 22, 35]. However, our in vitro findings did not correlate with observations in xenograft models, where FMOD deficiency did not affect angiogenesis, as indicated by the comparable densities of tumor-associated endothelial structures. This is consistent with a previous study in human anaplastic thyroid carcinoma, which found that FMOD was not implicated in vessel formation in vivo [42]. Finally, our finding was corroborated in human tumors where FMOD levels did not correlate with vascular density. Vascular hyperpermeability is present in advanced brain lesions but also in micrometastases [43]. Interestingly, we found that FMOD altered the integrity of the BBB and increased its permeability in vitro. This was consistent with findings in BrM patients where we found a positive correlation between FMOD levels and edema-to-tumor volume ratio, suggesting that FMOD may induce BBB leakiness. Since in our model FMOD can regulate adhesion to the endothelium, cell migration, and vascular permeability, we concluded that FMOD might promote BBB transmigration to facilitate the seeding of metastatic cells into the brain parenchyma.

SOX2 has distinct roles across diseases, and its correlation with clinical outcomes is variable [44]. SOX2 expression is essential for the maintenance of tumor-initiating cells, which display cancer stem cell-like characteristics [26, 45]. SOX2 promoted BrM in breast cancer [19]. Cancer cells show heterogeneity in intrinsic metastatic potential; only a small subset of cells accrue and maintain competency to establish metastasis and sustain growth. While SOX2 was expressed in the vast majority of Cl.2A cells, its staining pattern in patient tumors was heterogeneous, with only a minority of cells expressing SOX2. This pattern is consistent with SOX2 having a role in a distinct cell subpopulation such as “stem-like” cells [46]. Since SOX2 is a lineage-defining transcription factor, it seems plausible that we selected for or against a lineage-committed cell population, which might reflect the specific phenotype differences seen between our variant clones. In our model, loss of SOX2 did not affect BrM, suggesting that its upregulation might not be essential in this process. Validation in melanoma BrM patients failed to show a correlation between SOX2 expression and different clinical parameters. Paradoxically, dual SOX2 and FMOD depletion abolished BrM in our model, suggesting that SOX2 and FMOD cellular programs are closely coordinated. The clinical significance of this finding was highlighted by our analysis of patient tumors, which showed elevated levels of both molecules in BrMs relative to other sites. Moreover, upregulation of both FMOD and SOX2 was a risk factor for developing early BrM. Further analyses indicated that FMOD and SOX2 are not functionally equivalent and that, in addition to their redundant functions, they serve specific roles during BrM. However, our data suggest that SOX2 is dispensable, since FMOD could fully compensate for SOX2 absence. Hence, it seems plausible that FMOD function predominates in the nervous system, since FMOD depletion significantly delayed BrM with no significant effects extracranially. These observations underscore the complex interplay between FMOD and SOX2 in melanoma metastasis.

Like SOX2, FMOD is involved in cell fate determination, as it mediates cell reprogramming [44, 47, 48]. FMOD and SOX2 might both ensure cancer cell self-renewal capacity (stemness), a property linked to the induction of EMT and required for metastatic outgrowth [2]. The EMT program is a key regulator of niche activation, which plays essential roles in supporting the survival and self-renewal of disseminated cancer cells [2, 9]. Reversal of this state (MET) after disseminated cancer cells extravasate facilitates colonization and metastatic outgrowth [2]. Unlike FMOD and SOX2 depleted cells, aggressive control cells expressed key transcription factors that govern the EMT process and exhibited EMT characteristics. It is possible that FMOD and SOX2 contribute to confer a selective metastatic initiation advantage within the brain niche, by supporting stem cell functions and by promoting a more mesenchymal status and enhanced stromal niche activation capacity during the early phase of metastatic colonization.

The striking effects of dual FMOD and SOX2 silencing on metastasis suggest that FMOD-regulated tumor–host interactions might be coupled with SOX2-driven signaling mechanisms in our model. The significant alteration of proliferation and colony formation observed in vitro upon dual FMOD and SOX2 silencing, which contrasted the effects of single-gene depletion, suggests that FMOD and SOX2 functional roles might converge at the induction of tumor growth. Interestingly, variant cells showed similar tumor growth rates when grown subcutaneously. This suggests that FMOD and SOX2 might regulate metastatic competence without altering the tumorigenic properties and highlights their functional specificity in melanoma BrM. Studies show that tumor and stromal cells cooperate in forming distinct metastatic niches at different anatomical sites [4, 34, 49, 50]. An explanation of our finding is that the anti-tumor responses are regulated differently by the brain and subcutaneous microenvironments, with the latter being more permissive. We previously showed that the brain tumor microenvironment is unique and that BrMs are less vascularized compared to other extracranial sites including skin or soft tissues [17]. Nevertheless, BrMs can develop irrespective of angiogenesis through exploiting alternative vascularization mechanisms [51]. It is possible that Cl.2A cell variants gain increased support from the skin stroma and therefore display similar efficiencies in recruiting local endothelial cells to initiate angiogenesis to sustain growth subcutaneously. FMOD and SOX2 might confer a selective growth advantage, which is context dependent, hence contingent upon the presence of a distinct milieu found in the brain.

Aggressive variant cells readily induced vascular mimicry (VM) and lost this ability after both FMOD and SOX2 depletion. This contrasted with the effects of single-gene editing, which led to VM defects in FMOD knockouts but not SOX2. This raises the possibility that FMOD and SOX2 serve redundant roles in promoting VM, but their functions may not be equivalent as FMOD functions predominate. FMOD has yet to be linked to VM but SOX2 was shown to facilitate VM of “stem-like” cancer cells [27, 52]. VM is a form of neovascularization exploited by high-grade, rapidly growing, and aggressive tumors in critical need of increased blood supply due to their high metabolic demand [28]. Tumors formed by our model had the ability to stimulate traditional angiogenesis, while vascular density was comparable among cohorts. Interestingly, unlike brain lesions, subcutaneous xenografts generated by Cl.2A variants showed similar VM formation suggesting that microenvironmental differences probably accounted for this discrepancy. It is possible that Cl.2A tumors require additional support from atypical vasculature for adequate nutrient supply to self-sustain expansion in highly non-permissive, poorly vascularized tumor microenvironments found in the CNS. Collectively, our data suggest that FMOD and SOX2 are involved in VM, which might be exploited as an alternative vascularization mechanism and might be a rate-limiting step for the expansion of BrM in our model.

**Fig. 10:**
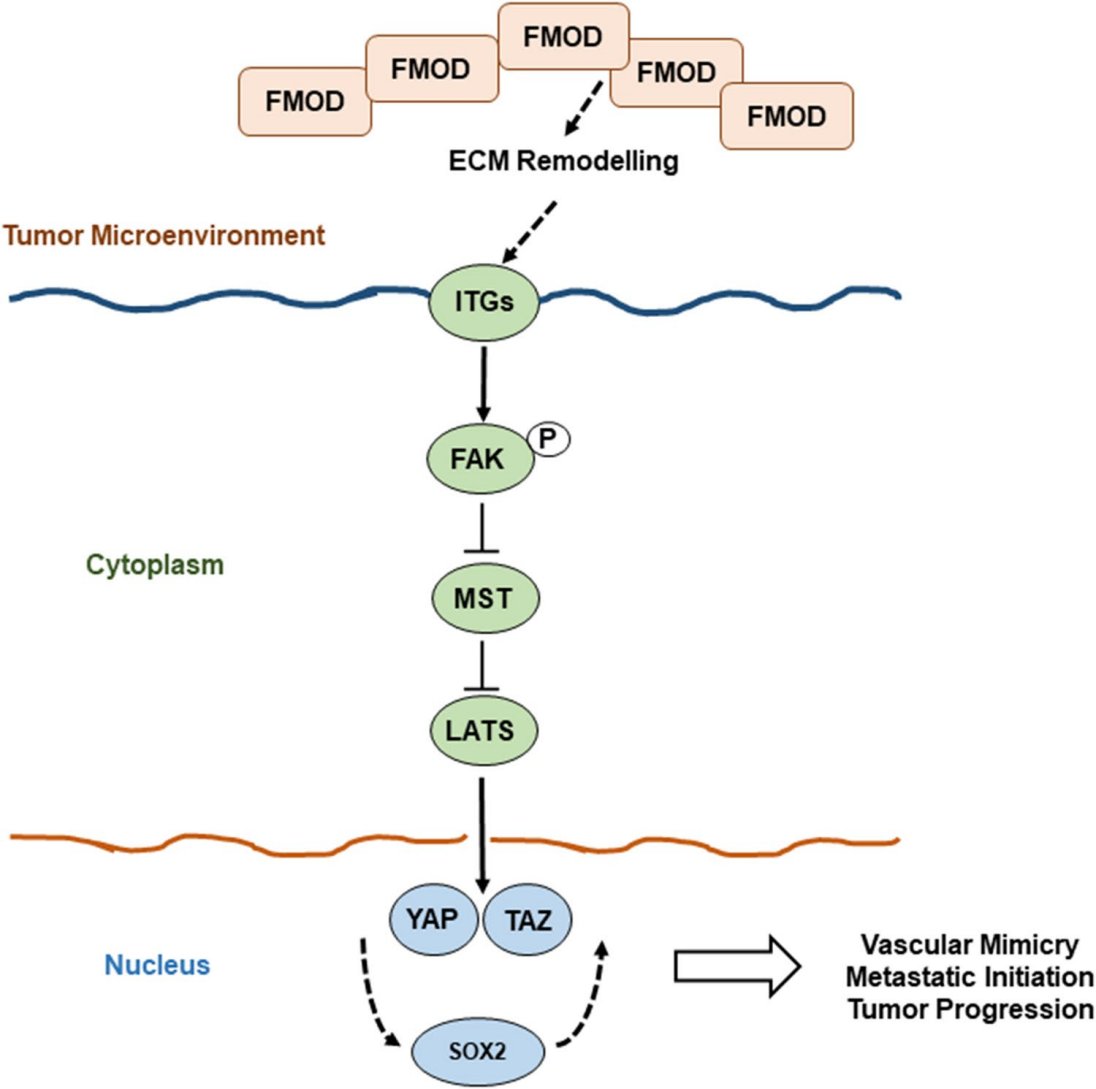
Graphical abstract of FMOD mediated brain metastasis in melanoma. High FMOD expression in the tumor microenvironment remodels the extracellular matrix activating the integrin/FAK pathway, which promotes YAP/TAZ nuclear translocation and subsequent initiation of metastatic outgrowth

Our data reveal a novel mechanism linking FMOD to the activation of transcriptional co-factors YAP and TAZ, possibly via integrin/FAK signaling and crosstalk with the Hippo tumor suppressor pathway (Fig. 10). While studies of YAP/TAZ in melanoma are limited, in other cancers, they were shown to regulate tumor initiation and cancer stemness and more recently found to play critical roles in angiogenesis and VM [27]. YAP/TAZ are primary intracellular sensors of ECM stiffness and remodeling as well as cytoskeletal induced mechanical changes [32]. Importantly, our transcriptome analysis showed that loss of FMOD was concomitantly associated with decreased expression of several genes encoding important ECM components such as POSTN, suggesting that FMOD functions may depend on the presence of specific ECM proteins. Interestingly, double knockout also led to downregulation of TNC gene. Periostin and tenascin-C bind to cell surface integrins and functionally cooperate to maintain metastatic niches [2]. Moreover, both molecules activate YAP/TAZ by interfering with integrin-FAK/Src signaling, which leads us to speculate that FMOD might not be the primary or sole mediator of microenvironmental stimuli to regulate YAP/TAZ [53, 54]. Judging by the marked increase in the levels of phosphorylated MST1, LATS1 and their downstream targets YAP and TAZ in FMOD/SOX2 depleted cells, compared to single-gene silenced variants, we suggest YAP/TAZ regulation as a point of convergence between FMOD and SOX2. In support of this hypothesis is our finding that several YAP/TAZ regulated genes known to induce EMT as well as VM were highly differentially expressed in the FMOD/SOX2-silenced cells compared to FMOD knockout or control cells [28]. Thus far, we have not investigated the molecular mechanisms linking SOX2 to YAP/TAZ activity, though studies attest that this cooperation exists. SOX2 antagonizes Hippo signaling to maintain cancer cell stemness, while YAP can regulate SOX2 to facilitate self-renewal and vascular mimicry of “stem-like” cancer cells [52, 55]. The downstream mechanisms of SOX2 action that regulate YAP/TAZ and the crosstalk with signaling pathways controlled by FMOD in our model remain to be explored.

The experimental models presented here are ideal preclinical tools to study factors regulating brain-metastatic outgrowth in melanoma, an area that remains largely unexplored. While our model is preferentially cerebrotropic allowing for studies of disease progression in the brain, independently of tumorigenic effects elsewhere, it has the added advantage of closely reflecting the clinical scenario, as it tends to induce extra-cerebral metastases, as well; the majority of melanoma patients with brain involvement have extra-cerebral metastases. Our finding that dual SOX and FMOD silencing abolishes BrM, with a similar effect on metastasis at extracranial sites, is likely important. Further investigation of the joint role of these molecules is warranted as it may provide novel avenues for therapeutic intervention for patients with microscopic metastasis at risk for developing overt stage IV disease. A limitation of our study is that conclusions are based on observations in one model system, and in nude mice. Efforts are ongoing in our laboratory to confirm these findings in additional clinically relevant xenograft and immune-competent models to establish the importance of this cooperation in melanoma metastasis and to understand the precise role of FMOD protein in engaging mechanical signals to sustain YAP/TAZ activity in metastasis.

## Supplementary Information

Below is the link to the electronic supplementary material.

Supplementary file1 (DOC 67 KB)

Supplementary Fig. 1. Role of FMOD knockout in Cl.2A cells. (A) Immunoblot of FMOD (conditioned medium) and SOX2 (cell lysate) expression in parental, Cl.1A, Cl.2A, and Cl.2B melanoma cells. (B, C) FMOD and SOX2 quantification of mRNA levels by qPCR (B) and by immunoblot (C) in Cl.2A cell variants. (D, E) Quantification of proliferation, apoptosis indices (D), and angiogenesis (E) in Cl.2A cells following loss of FMOD expression. Data analysis included at least two different regions per tumor per mouse. (F, G, H, I) Depletion of FMOD in Cl.2A cells did not affect cell proliferation both, under standard culture conditions, or starvation (F), spontaneous apoptosis (G), colony formation (H), or invasion through Matrigel matrix (I). Data are expressed as mean ± SD of three biological replicates and statistical significance determined using a two-sided Student t-test:*P<0.05, **P<0.01, ***P<0.001, and ****P<0.0001. Supplementary Fig. 2. Combined effect of FMOD and SOX2 knockout in Cl.2A cells. (A) Representative images of bioluminescence in mice 6 weeks post intracardiac injection with Cl.2A variants labeled with GFP and luciferase. (B) H&E staining of a double SOX2&FMOD knockout tumor showing dormant tumor cells and micrometastases. (C) Quantification of proliferation, apoptosis indices, and angiogenesis in Cl.2A cells following loss of SOX2 expression. Data analysis from at least two different regions per tumor per mouse. (D) Immunofluorescence staining of SOX2 expression reveals a distinct nuclear expression pattern in control cells compared to FMOD-/- cells. (E) Quantification of spontaneous apoptosis in Cl.2A following loss of SOX2 and SOX&FMOD. (F) Tumor growth rates of Cl.2A variants injected subcutaneously in athymic nude mice, monitored for five weeks. Data displayed are representatives of two independent experiments. (G) Dual loss of SOX2&FMOD has no additive impact on Cl.2A cell migration and (H) Matrigel matrix invasion. (I) Treatment of HUVECs with conditioned medium from Cl.2A cells depleted of SOX2 and/or FMOD had no effect on proliferation. (J) Dual SOX2 and FMOD depletion has no additive effect on HUVECs transmigration. (K) Quantitation of CD34+ and PAS+ vessels in subcutaneous xenografts from at least two different regions per tumor in each mouse. All data are expressed as mean ± SD of three biological replicates and statistical significance determined using a two-sided Student t-test:*P<0.05, **P<0.01, ***P<0.001, and ****P<0.0001. Supplementary Fig. 3. Analysis of FMOD expression in melanoma patient samples. (A) Regression plot demonstrating a strong correlation between FMOD expression in the stroma and the S100-postive tumor compartment. (B, C) Association between FMOD expression and tumor characteristics. ANOVA was used to compare FMOD expression (continuous intensity scores) in the stromal (B) or tumor compartments (C), among specimens of different origin. Brain and other visceral metastases had the highest FMOD expression. (D) Two sample test showing no significant association between FMOD expression (continuous FMOD intensity scores) in the stromal compartment and vascular density measured by CD34-positivity (scores dichotomized by the median value). (E) Two sample test showing that high FMOD expression (continuous FMOD intensity scores) in the stromal compartment was significantly associated with high edema-to-tumor volume ratio (scores dichotomized by the median value). (F, G) Kaplan–Meier curves showing no significant association between SOX2 expression and BMFS (F) or OS (G) (BR-brain, LN-lymph node, SK-skin, ST-soft tissue, and V-viscera) (PDF 484 KB)

Supplementary Table 1: List of significantly upregulated and downregulated genes comparing parental cell YUGEN8, metastatic indolent cell Cl.1A, and metastatic competent cells Cl.2A and Cl.2B (XLS 109 KB)

Supplementary Table 2: Table showing the percentages of brain and extracranial metastasis in Cl.2A variants (XLS 27 KB)

Supplementary Table 3: List of significantly upregulated and downregulated genes in Cl.2A cells following FMOD and SOX2 knockout (XLS 166 KB)

Supplementary Table 4: List of enriched functional categories in FMOD-/- and SOX2/FMOD-/- cells generated from the up/downregulated gene sets (XLS 27 KB)

Supplementary Table 5: List of dysregulated ECM proteins in FMOD-/- and SOX2/FMOD-/- Cl.2A cells (XLS 34 KB)

Supplementary Table 6: List of antibodies used in our study (XLS 38 KB)

## Data Availability

All microarray datasets described in this publication are accessible on the NCBI GEO platform through series accession numbers GSE183179 and GSE183180. All data generated or analyzed during this study are included in this article and its supplementary files, and accessible from the corresponding author upon reasonable request.

## Abbreviations

FMOD: Fibromodulin
SOX2: SRY (sex-determining region Y)-box 2
YAP: Yes-associated protein 1
TAZ: Transcriptional coactivator with PDZ-binding motif
VM: Vascular mimicry
BrM: Brain metastases
LV: Left ventricle
BLI: Bioluminescence imaging
TEER: Trans-endothelial electrical resistance
BBB: Blood–brain barrier
RLU: Relative luminescence units
RFU: Relative fluorescence units
