# Supplementary material for: Coupled fibromodulin and SOX2 signaling as a critical regulator of metastatic outgrowth in melanoma
Source: Cell Mol Life Sci. 2022 Jun 23;79(7):377. doi: 10.1007/s00018-022-04364-5 (PMC9226089; doi:10.1007/s00018-022-04364-5)
Supplement: Supplementary file 1 — Supplementary file1 (DOC 67 KB) [file 18_2022_4364_MOESM1_ESM.doc]

**Coupled fibromodulin and SOX2 signaling as a critical regulator of metastatic outgrowth in melanoma**

Victor O. Oria, Hongyi Zhang, Christopher R. Zito, Chetan K. Rane, Xian-Yong Ma, Olivia K. Provance, Thuy T. Tran, Adebowale Adeniran, Yuval Kluger, Mario Sznol, Marcus W. Bosenberg, Harriet M. Kluger, Lucia B. Jilaveanu*

***Corresponding Author**

lucia.jilaveanu@yale.edu

**Supplementary Methods**

**In vivo metastasis and tumor growth assays**

For metastasis assay, animals were anaesthetized followed by left ventricle injection of tumor cells (100,000 cells in 100 µl). Metastatic growth was monitored once a week by bioluminescence imaging using IVIS Spectrum. At respective end-points, mice were euthanized in a carbon dioxide chamber and brains were dissected for analysis. Experiments were repeated three times. Immunohistochemical staining of Ki67, TUNEL, CD34, and PAS was performed by Yale Pathology Tissue Services (YPTS). Analysis of apoptosis and proliferation indices was done using Image J. For CD34 and PAS staining, individual vessels per field were blindly counted and quantification was done by averaging the number of positively stained vessels from a minimum of two regions per tumor. For subcutaneous tumor growth, we injected 3 x 10^5^ cells in 100 µl of PBS: Matrigel (1:1 ratio) into both flanks of nude mice (n = 5 per group). Tumor growth was measured twice a week using a digital caliper and tumor volume estimated using the formula (V = L x (W^2^)/2).

**Lentiviral constructs and guide-RNAs**

The gRNA selected for *SOX2* gene (gSox2-1: ATTATAAATACCGGCCCCGG) is located in the middle of exon 1 located on chromosome 3. For *FMOD* gene, the following gRNAs were used: gFmod2: CCGTCCCCGATAGCTACTTC located downstream of exon 2, gFmod3: GAAGTTCACGACGTCCACCA located upstream of exon 3 and gFmod3B-B: GTCATAGGGATCGTAGTAGG located upstream of exon 2. The single gRNA with 5’ overhang BsmBI sites was synthesized and cloned into the Lenti-CRISPR v2 backbone under the hU6 promoter. The WT Cas9 nuclease expressed under the EFS promoter was purchased from GeneScript. The pCDH-EF1-Luc2-P2A-tdTomato was purchased from Addgene, with the Luc and tdTomato expressed under EF1a promoter. The lentivirus plasmids were packaged individually in immortalized human embryonic kidney cells HEK293T using the packaging plasmids pLP1, pLP2 and pLP-VSVG following the standard lentiviral production protocol.

**Transcriptomic profiling**

The intactness of the RNA was monitored by control probes to housekeeping genes. Data was considered accurate only when housekeeping gene values were similar for all arrays in one experiment. Negative controls, probes of random sequence selected to have no corresponding targets in the genomes, were included. The mean signal of these probes was utilized to define the system background. The raw expression data was first processed with R package oligo (1) normalized with robust multi-array analysis (RMA) (2) prior to downstream analysis. The log2 gene expression was comparable in all the 12 samples and demonstrated a high degree of standardization. We conducted hierarchical clustering and principal component analysis (PCA) to determine whether these samples displayed distinguishable transcriptome profiles. For comparative analyses between groups, we employed linear models for microarray data (LIMMA), an R-inbuilt package. This is powerful tool in omics data about multiple testing correction and prevention of false positive discoveries (3). As a cutoff to distinguish significantly regulated genes, we chose a false discovery rate (FDR) value of 0.05 and a fold change value of 2.0 (log2 FC of 1.00 and -1.00). For subsequent downstream experiments, we choose Cl.2A as a model to study BrM initiation in melanoma.. To investigate the effect of FMOD, SOX2, and SOX2&FMOD silencing on Cl.2A cells, we conducted transcriptomic profiling using Human Clariom^TM^ S array (Thermo) that can accurately quantify gene-level expression differences from over 20,000 well-annotated genes. Analysis of microarray data was performed as described above and datasets described in this publications accessible through GEO series accession numbers GSE183179 and GSE183180.

**Cell cycle analysis**

Cells were initially sub-cultured for at least 24 hours followed by synchronization into G1/S-phase by double-thymidine block. Sample preparation and flow cytometry analysis were performed as previously described (4).

**Cell migration and invasion**

Tumor cell migration was investigate using the gap closure assay as previously described (5). *In vitro* tumor cell invasion and endothelial cell transmigration were evaluated using the Corning FluoroBlok Tumor Invasion System (Corning Incorporated) and endothelial cell migration assay (ECM200: Millipore) respectively following the manufacturer’s protocol.

**Cell Adhesion assay**

Cell adhesion assay was done as previously described (5) using a colorimetric ECM Cell Adhesion Array Kit (ECM540: Millipore).

**Spontaneous Apoptosis and Cell viability**

Spontaneous apoptosis was evaluated using annexin V-Alexa Fluor 488 (Thermo) and propidium iodide (Thermo) staining according to manufacturer’s instructions. Flow cytometry was performed with a FACScalibur (BD Biosciences), and results were analyzed with FlowJo software (Tree Star, Inc).

The impact of the loss of FMOD and SOX2 on Cl.2A cytotoxicity was assessed using the MTT assay. 2 x 10^5^ cell per well were seeded in a 48-well plate and treated with 20 µM etoposide, a known inducer of cytotoxicity, for 72 hours. 100 µl of MTT solution and serum free medium (1:1 ratio) was added into each well and incubated for 3 hours. 150 µl of MTT solvent was added into each well, incubated for 5 minutes, and absorbance read at 590 nm. Values of treated cells grown in the presence of etoposide were compared with untreated cells under the same conditions and reported as percent cytotoxicity. Experiments were done under standard culture and starvation conditions.

**Immunoblotting**

For immunoblotting experiments, c**ells lysis was done using NP40 solution supplemented with 1mM Na_3_VO_4_, 1mM PMSF** containing the protease inhibitor cocktail. Protein concentrations of lysates was determined Bicinchoninic Acid (BCA) assay. 30 µg of protein was diluted in a sample buffer (4X Laemmli Buffer: Bio-Rad supplemented with 1 part β-mercaptoethanol) and subjected to sodium dodecyl sulfate-polyacrylamide gel electrophoresis (SDS-PAGE). **The list of primary antibodies used are in the Supplementary Table 6.** Detection of proteins was done with HRP-conjugated anti-mouse or anti-rabbit IgG secondary antibodies (#7076 and #7074 respectively: Cell Signaling). Proteins were detected and quantified in three biological replicates per cell line.

**Quantitative RT-PCR**

Total RNA was extracted from cultured cells using RNeasy Kit (Qiagen) followed by reverse transcription using the iScript™ cDNA Synthesis Kit (Bio-Rad). Quantitative PCR was performed using Power SYBR® Green PCR Master Mix (Applied Biosystem/Thermo Fisher Scientific, Life Technologies). β-actin levels were used as an endogenous control for normalization and data analysis was performed using the comparative threshold cycle (CT) method. The following primers were used:

| **Gene** | **Forward Primer** | **Reverse Primer** |
| --- | --- | --- |
| ACTIN | AGCACTGTGTTGGCGTACAG | CTCTTCCAGCCTTCCTTCCT |
| BIRC5 | GGACCACCGCATCTCTACATT | TGTTCCTCTATGGGGTCGTCA |
| BMP4 | ACCGAATGCTGATGGTCGTT | TCTGCTCTTCCTCCTCCTCC |
| CDC20 | GTAGGCACCAACTGCAAGGA | CTGCGATGGGGGATATAGCG |
| FMOD | AAGTACCTGCCCTTCGTTCC | CCTGCCCACCTTATCACTGG |
| SOX2 | CATGAAGGAGCACCCGGATT | ATGTGCGCGTAACTGTCCAT |
| ZEB2 | CAGCTAGTGTGCCCAACCAT | TAATTGCGGTCTGGATCGTGG |

**Tissue microarray construction and immunofluorescent staining**

Briefly, slides were deparaffinized in xylene followed by three rinses in 100% ethanol. Antigen retrieval was done by boiling the slides in 6.5mM sodium citrate (pH 6.0) under pressure followed by incubation in a mixture of methanol and 2.5% hydrogen peroxide for 30 minutes at room temperature to block the endogenous peroxidase activity. To block non-specific staining, slides were incubated in 0.3% bovine serum albumin/1X Tris-buffered saline followed by overnight incubation at 4°C overnight with anti-FMOD antibody (sc-166406: Santa Cruz). Slides were stained with anti-S100 antibody (GA504: Dako) to create a tumor mask and localize the cell membrane/cytoplasmic compartment within this tumor mask. The FMOD signal was amplified by goat anti-rabbit horseradish peroxidase-decorated polymer backbone (Envision, Dako) and the staining was visualized with Cy5-tyramide (NED Life Science Products). A secondary goat IgG conjugated to Alexa 546 was used for visualization of the S100 signal (Molecular Probes, Inc.). The nuclear compartment with the tumor mask was identified by mounting the coverslips with ProLong Gold antifade reagent containing 4', 6-diamidino-2-phenylindole (DAPI) (Invitrogen). Automated image acquisition and analysis using JMP 5.0 software (SAS Institute) was conducted as previously described (6). Data was analyzed using either continuous immunofluorescence scores or variables dichotomized by the median value. The association between continuous QIF scores and clinicopathological parameters was evaluated by the two-sample t test (analysis of variance, ANOVA). The Chi Square test was used for analysis of dichotomized variables. The prognostic significance was assessed using the Cox proportional hazards model with survival as an end point. Survival curves were generated using the Kaplan-Meier method.

**Supplementary References**

1. Carvalho BS, Irizarry RA (2010) A framework for oligonucleotide microarray

preprocessing. Bioinformatics 26(19):2363-7.

2. Irizarry RA, Bolstad BM, Collin F, Cope LM, Hobbs B, Speed TP. (2003) Summaries of

Affymetrix GeneChip probe level data. Nucleic Acids Res 31(4):e15.

3. Ritchie ME, Phipson B, Wu D, Hu Y, Law CW, Shi W, et al. (2015) Limma powers

differential expression analyses for RNA-sequencing and microarray studies. Nucleic

Acids Res 43(7):e47.

4. Zhang H, Zhu H, Deng G, Zito CR, Oria VO, Rane CK, et al. (2020) PLEKHA5

regulates tumor growth in metastatic melanoma. Cancer 126(5):1016-30.

5. Oria VO, Lopatta P, Schmitz T, Preca BT, Nystrom A, Conrad C, et al. (2019) ADAM9

contributes to vascular invasion in pancreatic ductal adenocarcinoma. Mol Oncol

13(2):456-79.

6. Camp RL, Chung GG, Rimm DL. (2002) Automated subcellular localization and

quantification of protein expression in tissue microarrays. Nat Med 8(11):1323-7.
