# Supplementary material for: Coupled fibromodulin and SOX2 signaling as a critical regulator of metastatic outgrowth in melanoma
Source: Cell Mol Life Sci. 2022 Jun 23;79(7):377. doi: 10.1007/s00018-022-04364-5 (PMC9226089; doi:10.1007/s00018-022-04364-5)

# Coupled fibromodulin and SOX2 signaling as a critical regulator of metastatic outgrowth in melanoma

Victor O. Oria, Hongyi Zhang, Christopher R. Zito, Chetan K. Rane, Xian-Yong Ma, Olivia K. Provance, Thuy T. Tran, Adebowale Adeniran, Yuval Kluger, Mario Sznol, Marcus W. Bosenberg, Harriet M. Kluger, **Lucia B. Jilaveanu***

***Corresponding Author**
lucia.jilaveanu@yale.edu

## List of Supplementary Figures

**Supplementary Fig. 1**

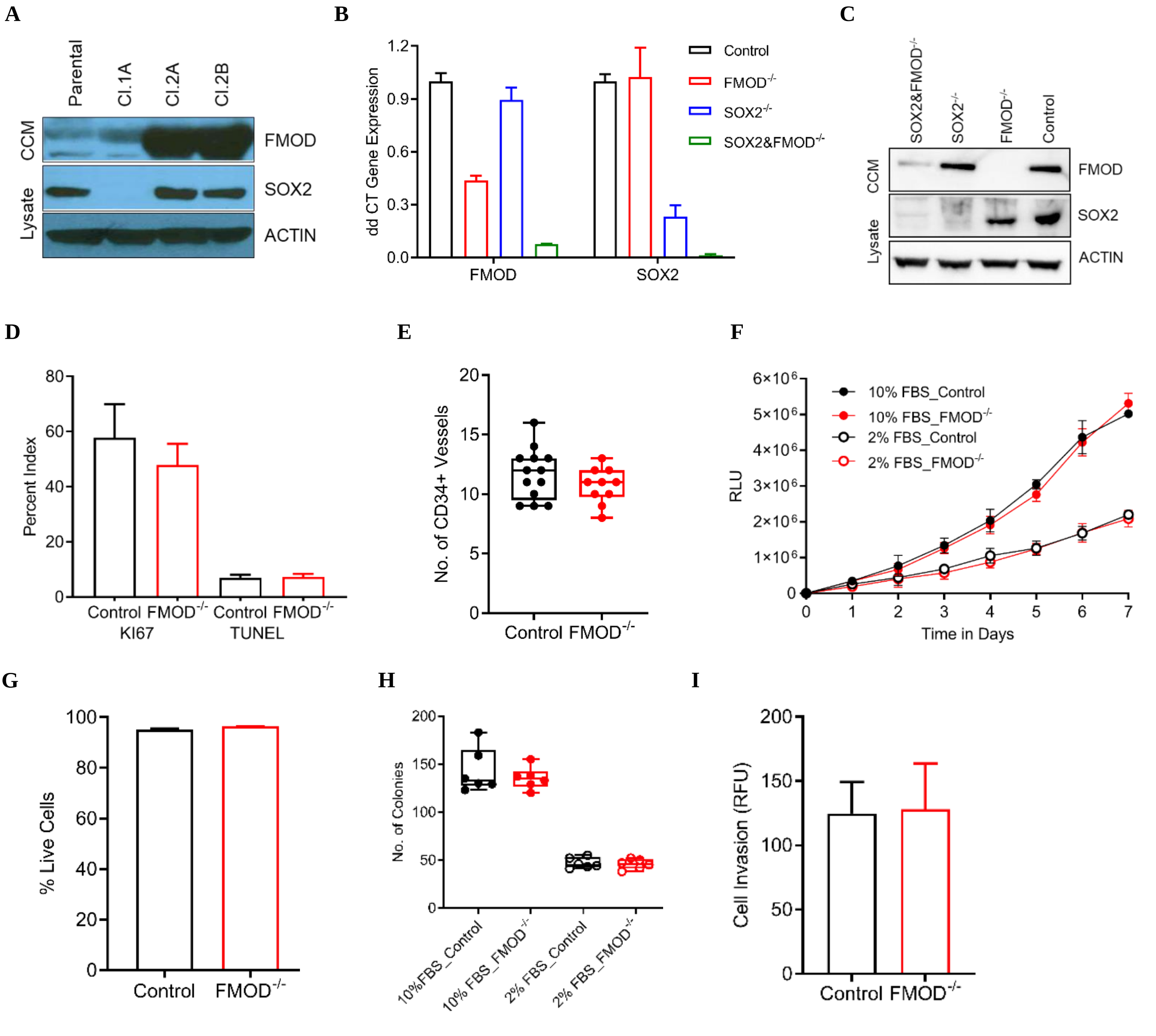

## Supplementary Fig. 2

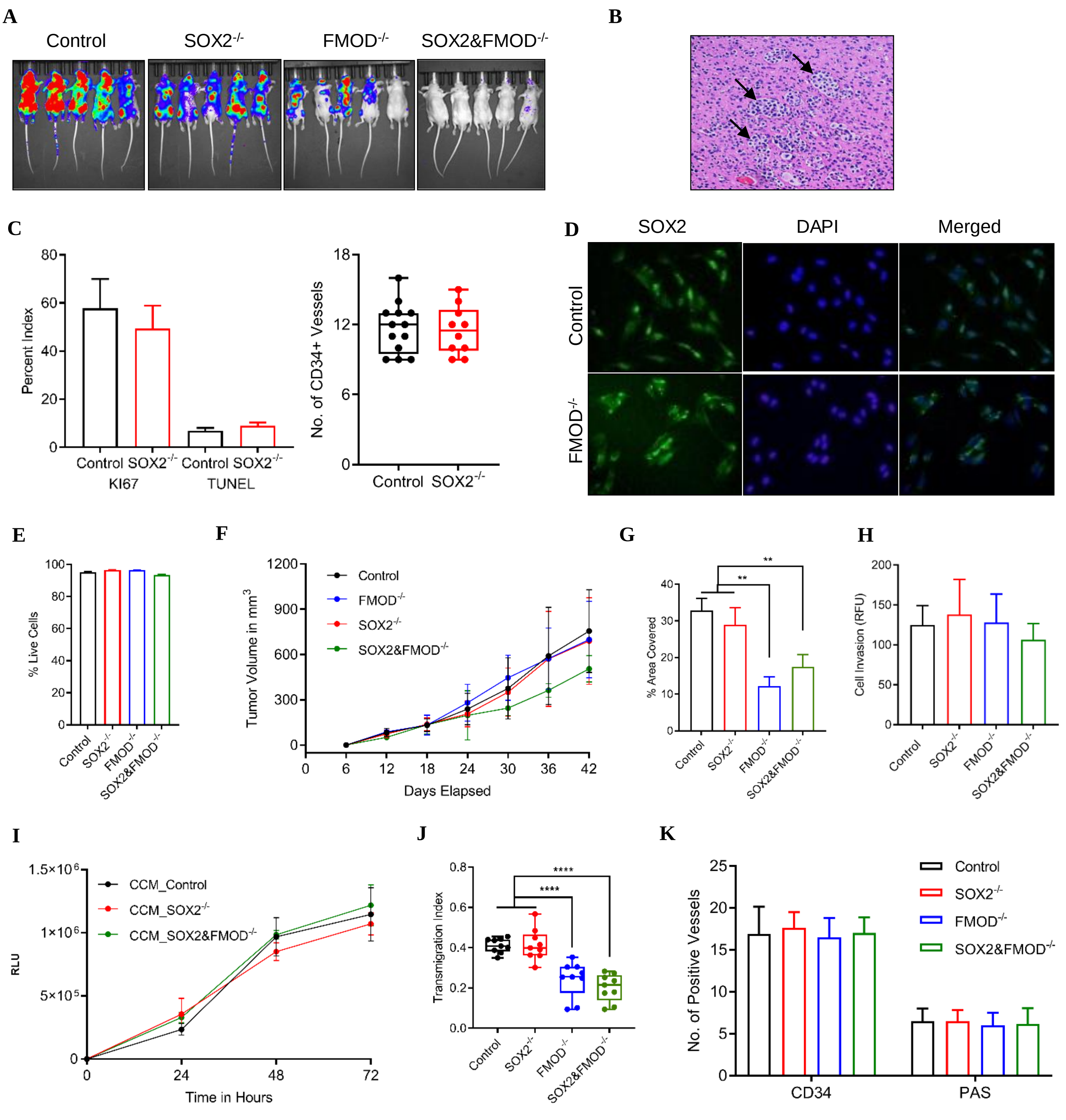

**Supplementary Fig. 3**

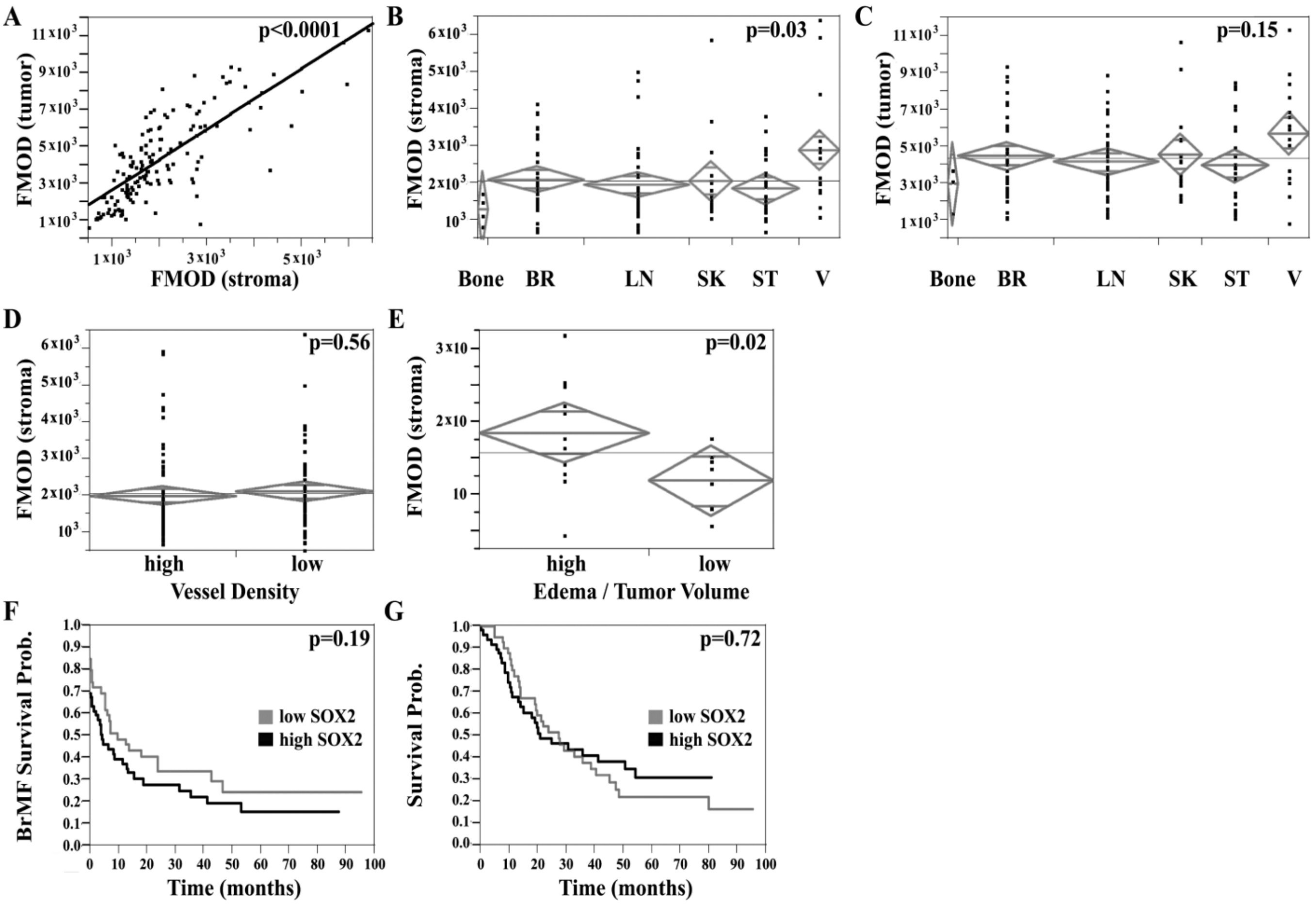

Supplement: Supplementary file 2 — Supplementary Fig. 1. Role of FMOD knockout in Cl.2A cells. (A) Immunoblot of FMOD (conditioned medium) and SOX2 (cell lysate) expression in parental, Cl.1A, Cl.2A, and Cl.2B melanoma cells. (B, C) FMOD and SOX2 quantification of mRNA levels by qPCR (B) and by immunoblot (C) in Cl.2A cell variants. (D, E) Quantification of proliferation, apoptosis indices (D), and angiogenesis (E) in Cl.2A cells following loss of FMOD expression. Data analysis included at least two different regions per tumor per mouse. (F, G, H, I) Depletion of FMOD in Cl.2A cells did not affect cell proliferation both, under standard culture conditions, or starvation (F), spontaneous apoptosis (G), colony formation (H), or invasion through Matrigel matrix (I). Data are expressed as mean ± SD of three biological replicates and statistical significance determined using a two-sided Student t-test:*P<0.05, **P<0.01, ***P<0.001, and ****P<0.0001. Supplementary Fig. 2. Combined effect of FMOD and SOX2 knockout in Cl.2A cells. (A) Representative images of bioluminescence in mice 6 weeks post intracardiac injection with Cl.2A variants labeled with GFP and luciferase. (B) H&E staining of a double SOX2&FMOD knockout tumor showing dormant tumor cells and micrometastases. (C) Quantification of proliferation, apoptosis indices, and angiogenesis in Cl.2A cells following loss of SOX2 expression. Data analysis from at least two different regions per tumor per mouse. (D) Immunofluorescence staining of SOX2 expression reveals a distinct nuclear expression pattern in control cells compared to FMOD-/- cells. (E) Quantification of spontaneous apoptosis in Cl.2A following loss of SOX2 and SOX&FMOD. (F) Tumor growth rates of Cl.2A variants injected subcutaneously in athymic nude mice, monitored for five weeks. Data displayed are representatives of two independent experiments. (G) Dual loss of SOX2&FMOD has no additive impact on Cl.2A cell migration and (H) Matrigel matrix invasion. (I) Treatment of HUVECs with condition [file 18_2022_4364_MOESM2_ESM.pdf]
